# Inferring the regulatory network of the miRNA-mediated response to biotic and abiotic stress in melon

**DOI:** 10.1186/s12870-019-1679-0

**Published:** 2019-02-18

**Authors:** Alejandro Sanz-Carbonell, María Carmen Marques, Antonio Bustamante, Mario A. Fares, Guillermo Rodrigo, Gustavo Gomez

**Affiliations:** 1 Institute for Integrative Systems Biology (I2SysBio), Consejo Superior de Investigaciones Científicas (CSIC) - Universitat de València (UV), Parc Científic, Cat. Agustín Escardino 9, 46980 Paterna, Spain; 2 Instituto de Biología Molecular y Celular de Plantas (IBMCP), Consejo Superior de Investigaciones Científicas (CSIC) - Universidad Politécnica de Valencia (UPV), CPI 8E, Av. de los Naranjos s/n, 46022 Valencia, Spain; 3 Instituto Nacional de Investigaciones Agropecuarias (INIAP), Estación Experimental Pichilingue, Km5 vía Quevedo El Empalme, Mocache, Ecuador

**Keywords:** Agriculture, Climatic change, Cucurbits, Non-coding RNAs, RNA silencing, Stress tolerance

## Abstract

**Background:**

MiRNAs have emerged as key regulators of stress response in plants, suggesting their potential as candidates for knock-in/out to improve stress tolerance in agricultural crops. Although diverse assays have been performed, systematic and detailed studies of miRNA expression and function during exposure to multiple environments in crops are limited.

**Results:**

Here, we present such pioneering analysis in melon plants in response to seven biotic and abiotic stress conditions. Deep-sequencing and computational approaches have identified twenty-four known miRNAs whose expression was significantly altered under at least one stress condition, observing that down-regulation was preponderant. Additionally, miRNA function was characterized by high scale degradome assays and quantitative RNA measurements over the intended target mRNAs, providing mechanistic insight. Clustering analysis provided evidence that eight miRNAs showed a broad response range under the stress conditions analyzed, whereas another eight miRNAs displayed a narrow response range. Transcription factors were predominantly targeted by stress-responsive miRNAs in melon. Furthermore, our results show that the miRNAs that are down-regulated upon stress predominantly have as targets genes that are known to participate in the stress response by the plant, whereas the miRNAs that are up-regulated control genes linked to development.

**Conclusion:**

Altogether, this high-resolution analysis of miRNA-target interactions, combining experimental and computational work, Illustrates the close interplay between miRNAs and the response to diverse environmental conditions, in melon.

**Electronic supplementary material:**

The online version of this article (10.1186/s12870-019-1679-0) contains supplementary material, which is available to authorized users.

## Background

Any external factor that imposes a negative impact on plant growth and development is recognized as a stress inducer. Because of their sessile nature, plants have evolved sophisticated and robust mechanisms to cope with stress [1–5]. In general, plants respond to environmental alterations through a specific and well-defined reprogramming of their transcriptional activities with the aim of limiting the impact on their physiological states. However, it is well-known that, despite these regulatory mechanisms, the environment significantly affects plant survival, growth, and yield [1, 6]. Consequently, and boosted by the increase in world population and the corresponding alimentary demand, focused efforts are required for the improvement of crop plants to ensure sustainable food production [7].

The advent of different *omic* technologies has led to the identification and functional characterization of thousands of genes that are transcriptionally altered in response to stress (biotic and abiotic), suggesting their involvement in the maintenance of stress tolerance [8–10]. Such transcriptional alterations have revealed complex regulatory networks that underlie the responses to environmental stimuli. The elucidation of such networks is hence pivotal for a better understanding of the molecular mechanisms that enable plants to respond and eventually adapt to the environment [11].

The mechanistic and quantitative aspects involved in the fine regulation of stress-related genes, as well as in the regulatory networks that coordinate the recovery of homeostasis in a plant exposed to stress, remain hitherto to be deciphered [1, 12]. Increasing evidence collected from analyses of microRNAs (miRNAs) in diverse species (arabidopsis, rice, maize, sorghum, sunflower, etc.) subjected to stress has led to the general view that these miRNAs might play a key role in regulating the stress response in plants [1, 13, 14]. In this regard, more work is needed to determine how these miRNA-mediated regulatory pathways ultimately control gene expression and functionally connect plant responses with environmental changes [13, 15].

In plants, miRNAs are encoded by endogenous genes transcribed by RNA polymerase II (Pol II) to miRNA precursors that fold into stem-loop secondary structures. The hairpins are cleaved by the DICER-LIKE 1 (DCL1) protein in a duplex (normally of 21 or 22 nt in length) that is loaded into the ARGONAUTE 1 (AGO1) protein known to be part of the RNA-induced silencing complex (RISC) [15, 16]. Functionally, miRNAs regulate gene expression at the post-transcriptional level by inducing cleavage or translational repression of their mRNA targets (16; 17). In plants, only a few annotated miRNA gene families are highly conserved from mosses to higher flowering plants, whereas the majority are species- or family-specific, suggesting that most of the known miRNA genes arose relatively recently during evolution [17–20]. Currently, the general belief is that both common and specific miRNAs contribute to the coordinated regulation of the stress response in plants [1, 12, 20, 21].

Stress-responsive miRNAs have been described in plants exposed to both biotic (e.g., bacterial, fungi, and viral pathogenesis; [22–24]) and abiotic stresses (e.g., drought, salinity, nutrient deprivation, cold, or high temperature; [2, 12]). These seminal studies have shown that miRNAs may respond to the environment in a stress-, tissue-, and genotype-dependent manner. However, because of their derivation from a common ancestor, most plants share core gene networks that control the plant response to a wide range of environmental factors [1]. Consequently, it seems acceptable that the analysis of the responsiveness (considering equivalently both up- and down- directions) of conserved miRNAs to different stress conditions may help us to identify general and/or taxon-related mechanisms modulating the plant-environment interaction mediated by miRNAs. Furthermore, studies conducting systematic analyses of miRNA expression during controlled exposure to various environmental conditions at identical plant developmental stages are lacking [1]. Conducting such studies is, in our view, instrumental in order to infer robust regulatory networks underlying the common response to stress modulated by miRNAs. Finally, this is important for enhancing the knowledge gathered in model plants (e.g., *Arabidopsis thaliana*) with new results obtained in agricultural crops, bearing in mind the ultimate goal of improving crop protection and sensitivity [1].

Here, we use deep-sequencing, quantitative PCR, high scale degradome assays, and computational approaches to present the first comprehensive analysis of miRNA-target expression profiles in response to multiple abiotic (cold, drought, salinity, and short-day) and biotic (viroid infection, agrobacterium infiltration, and ascomycete root infection) stress conditions in melon. The general aim of this work was to identify stress-responsive miRNAs from melon, explore their roles in plant adaptation to changing environmental conditions, and infer the miRNA-mediated regulatory network of stress response in melon. To generate a robust knowledge suitable to be transferred to diverse plant species, only known miRNAs with well-established regulatory roles were considered. We chose melon for our research because it is a crop plant of great economic importance that is extensively cultivated in semi-arid regions [25]. In addition, diverse molecular tools have been developed for melon in the last few years, such as EST collections [26], TILLING platforms [27], and genome sequencing [28], which indeed favor the use of melon as a valuable experimental system to conduct investigations relevant for agriculture. Regarding the stress conditions analyzed, besides abiotic conditions well established as crucial for plant development (cold, drought, salinity and short-day) we select *Monosporascus cannonballus* (a soil borne fungal pathogen capable of causing root rot and wilting in melon; [29])-, *Hop stunt viroid* (a polyphagous pathogenic long ncRNA that is able to infect a wide range of hosts including cucurbits; [30]), and *Agrobacterium tumefasciens* (a plant-invasive bacteria frequently used in molecular biology), as biotic inducers of stress.

## Results

### Analysis of the sRNA population

We performed high-throughput sequencing (HiSeq 2000) of sRNA libraries constructed from leaves of plants at 11 days after exposure to seven stress conditions: a) cold, b) drought, c) salinity, d) short day, infection with e) *Monosporascus cannonballus* and f) *Hop Stunt Viroid* (HSVd), and g) infiltration with *A. tumefaciens*. Non-treated plants were used as controls (Additional file 1: Figure S1). Only sequences with size ranging between 20 to 25 nt in length were used in subsequent analyses. Reads fully homologous to rRNA, tRNA, snoRNA and snRNA sequences deposited in the Rfam data base (http://rfam.xfam.org), representing less of the 3.5% of the sequenced sRNAs (Additional file 2: Figure S2a), were filtered out. Under these conditions a total of 86,672,161 sRNA reads were recovered from control and treated plants, distributed by treatments as follows: *Agrobacterium*: 2,706,565 reads; cold: 11,583,717; control: 14,113,325; drought: 9,298,377; HSVd: 11,568,442; *Monosporascus*: 11,3393,625; salinity: 11,099,416; and short day: 11,055,876 (more details in Additional file 3: Table S1).

**Fig. 1:**
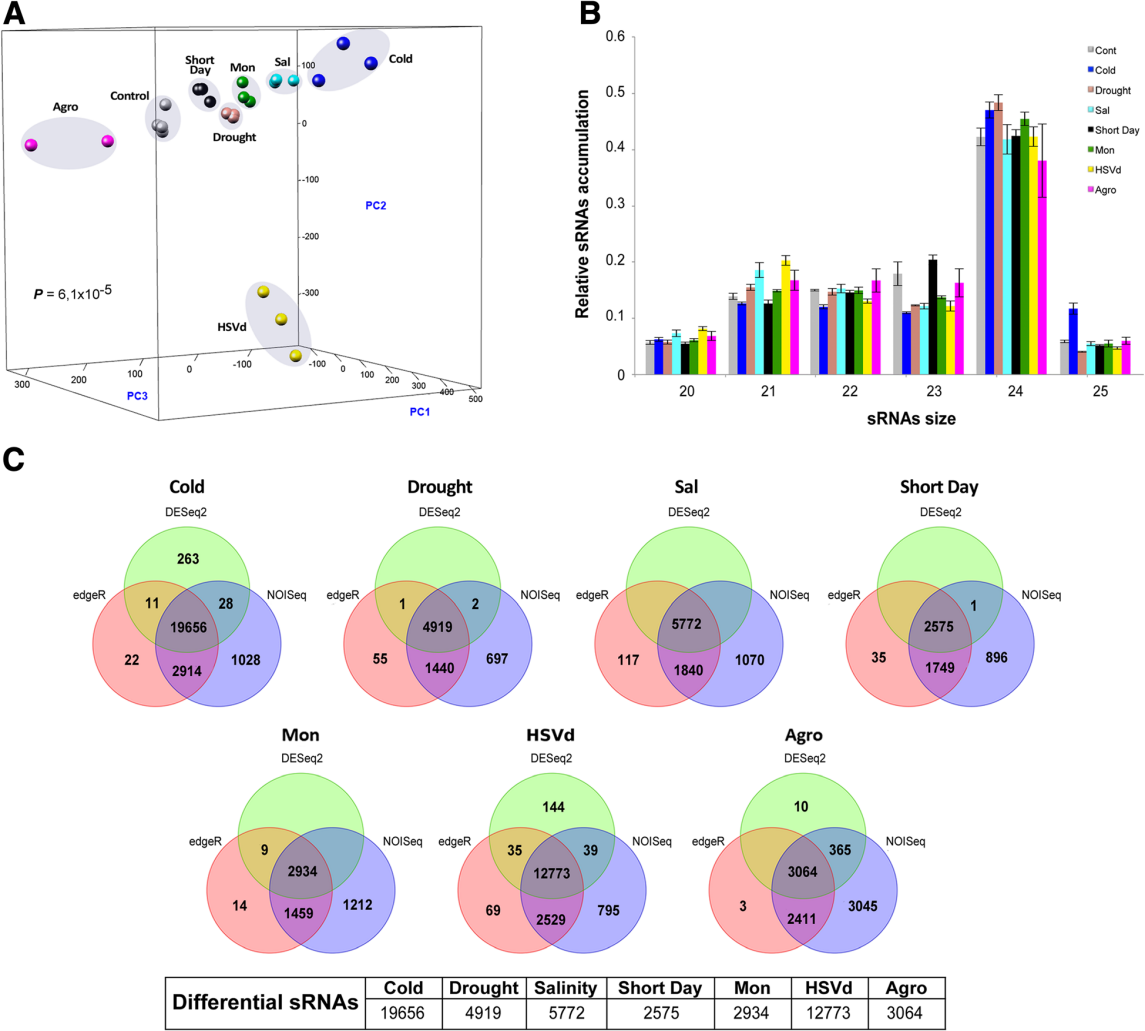
Analysis of the sRNA population recovered from analyzed melon libraries. **a** Principal component analysis based on sRNAs accumulation in replicates of melon-plants exposed to diverse stress treatments. The statistical significance was estimated by Mann-Whitney-Wilcoxon test, considering the inter- and intra-group Euclidean distances. **b** Diagram showing the relative accumulation (and distribution of the total clean reads of melon sRNAs ranging between 20 and 25 nt obtained from the 24 sequenced libraries. The control and the different analyzed treatments are represented with colors. The shown values represent the sum of all repetitions. **c** Venn diagram comparing the number of the differential sRNAs -estimated by DESeq2 (green), edgeR (red) and NOIseq (blue)- expressed in melon in response to cold, drought, salinity, Short Day, *Monosporascus* (Mon), HSVd and *Agrobacterium* (Agro) treatments. Only the sRNAs predicted as differential by all three analysis methods were considered as true stress-responsive miRNAs

Correlations between sRNA expression profiles (considering the different treatments and their biological replicates) were estimated by principal component analysis (PCA). The PCA plot shows that biological replicates of the same condition are clustered together with statistical significance (*P-*value = 6.15·10^− 5^), distinguished from other treatments, which attests the reproducibility of our assays (Fig. 1a). The proportions of variance explained by the three first components (PC1–3) were 26.38, 13.67, and 9.41%, respectively (cumulative proportion of 49.46%).

The sRNA dataset exhibited a distribution of read lengths enriched for 24 nt-long sequences (~ 43% of all reads), followed by a comparable accumulation of 21, 22, and 23 nt sizes (Fig. 1b). A slight increase in the proportion (~ 54%) of 24 nt-long reads was observed when unique sequences were analyzed (Additional file 2: Figure S2b). Our observation was consistent with total sRNA profiles previously described for leaves of *C. melo* [31, 32] and pooled leaves and fruit tissues of four members of the family *Cucurbitaceae* in which 24 nt-long sequences were over-represented [33]. Finally, and considering the general size profile of sRNAs, no obvious differences were observed between control and samples exposed to stress.

To evaluate the effects of stress on sRNA populations, we performed pairwise comparisons among control and treated samples with three statistical testing methods: edgeR, DESeq2 and NOISeq (criteria described in Material and Methods). Only the sequences predicted as differentially expressed by the three analysis tools were considered as true stress-responsive sRNAs. A total of 51,693 unique reads were identified as differentially expressed in melon plants exposed to the adverse conditions here analyzed (Fig. 1c). The low temperature treatment and HSVd infection induced the most drastic alterations in sRNA transcription (19,656 and 12,773 stress-responsive sequences), whereas this process was less disturbed in plants grown under short-day conditions or infected with *Monosporascus* (2575 and 2934 altered sRNAs, respectively). These results provided evidence that, coincident with observations in other analyzed plants, biosynthesis of sRNAs is sensitive to stress conditions in melon.

### miRNAs are generally down-regulated when responding to stress in melon

To identify miRNAs significantly altered in response to stress, sRNAs that exhibited differential accumulation (stress vs. control conditions) were aligned against the mature miRNAs sequence in the miRNA database [34]. Only fully homologous sRNAs were considered. Reads with an abundance value below the median of the base mean value for each miRNA family under each analyzed stress condition, were filtered out. Under these strict selection conditions we obtained 67 sequences belonging to 24 different known miRNA families (Additional file 4: Table S2 and Additional file 5: Figure S3).

**Fig. 2:**
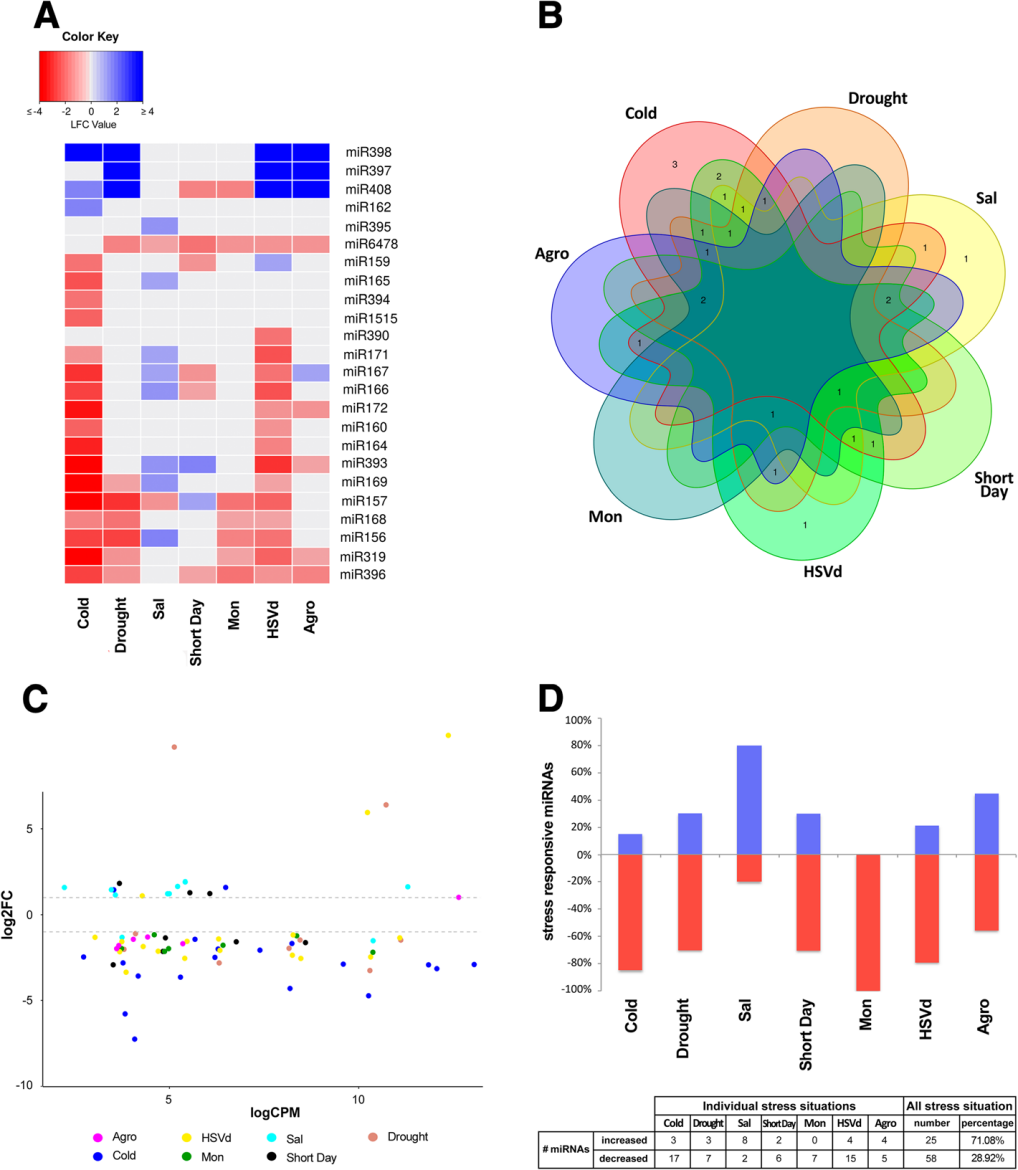
Analysis of stress-responsive miRNAs. **a** Heat map of 24 miRNAs differentially expressed in melon plants in response to stress. The differential expression values represented correspond to the median of the log2FC values obtained by DESeq2 analysis (detailed in the Fig. S5) in each miRNA family. **b** Venn diagram representing the number of the stress-responsive miRNAs expressed in melon plants exposed to cold, drought, salinity, Short Day, *Monosporascus* (Mon), HSVd and *Agrobacterium* (Agro) treatments. **c** Graphic representation of miRNA families significantly up- and down- regulated in melon plants exposed to different stress conditions. The dots indicate the expression value of the most representative sequence of each miRNA family identified as differentially expressed. The dot colors represent the different treatments. **d** Chart showing the relative accumulation (in percentage) of melon miRNAs up- (blue bars) and down- regulated (red bars) in plants exposed to different treatments. **e** Summary of the number of miRNA families with increased or decreased expression in response to stress

These miRNAs were identified in this work as stress-responsive miRNAs in melon (Fig. 2a, b). None of the 24 stress-responsive miRNAs showed significant differential accumulation under all seven conditions analyzed. However, four miRNAs (miR408, miR396, miR157, and miR6498) were differentially recovered from samples exposed to six of the stress conditions (Fig. 2a, b).

A high proportion of stress-responsive miRNAs (71.08%) was down-regulated (Fig. 2c, d and Additional file 6: Figure S4). All family-related sequences shown a comparable direction of accumulation (Additional file 7: Figure S5), supporting that, at least under the analyzed conditions, the response to stress in melon plants is common for different miRNA family members. The heat map of the differentially expressed miRNAs revealed that a decrease in the miRNA population was the general response to stress. Exceptionally, the miR397, miR408, and miR398 families showed higher accumulation in plants exposed to cold, drought, HSVd, and *Agrobacterium* than in control plants.

**Fig. 3:**
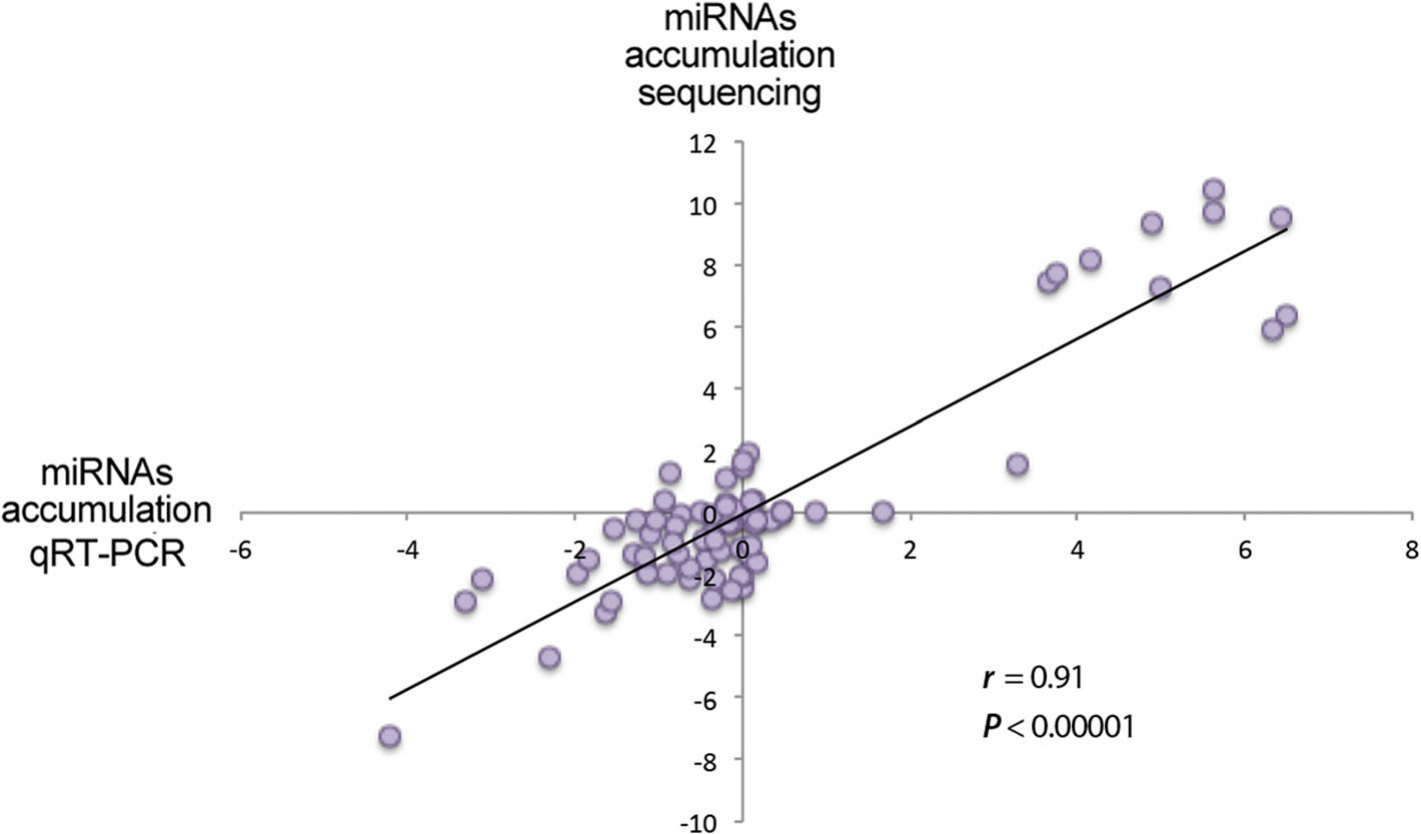
Validation of sequencing data by stem-loop qRT-PCR assay. Scatter plot showing the significant positive correlation (estimated by Pearson correlation coefficient) between the accumulation data obtained by sequencing and qRT-PCR for 6 representative miRNAs in all analyzed stress conditions (detailed information in Additional file 9: Table S3)

Considering each stress situation analyzed, we identified 20 miRNAs with significantly altered expression in response to cold, 10 to drought and salinity, 8 to short day, 9 to *Agrobacterium*, 7 to *Monosporascus*, and 19 to HSVd. As shown in Fig. 2c, where the specific expression level estimated for each miRNAs and stress condition is detailed, the most important response to stress associated with miRNAs was found in plants exposed to cold and HSVd. Among them, 17 miRNAs were down-regulated, and only three (miR398, miR408, and miR162) were significantly up-regulated by low temperature. On the other hand, and as consequence of HSVd infection, 15 and 4 miRNAs were down- and up-regulated, respectively. In contrast to the general response observed for the analyzed conditions, 8 out of 10 stress-responsive miRNAs observed in melon plants subjected to LiCl treatment showed increased expression (Fig. 2d). These sequencing-based profiling data were validated by estimation of accumulation by stem-loop qRT-PCR of twelve representative miRNA families. We found a significant correlation between the two techniques, mapping differential (lower and upper) and non-differential accumulation of miRNAs in response to multiple stresses (Fig. 3, Additional file 8: Figure S6 and Additional file 9: Table S3).

### Melon miRNAs respond predominantly in a stress-dependent manner

Subsequently, we inspected the particular expression profile of each miRNA. Many of the stress-responsive miRNAs (miR171, miR159, miR165, miR393, miR169, miR157, miR156, miR408, miR166, and miR167) exhibited a non-monotonous expression profile, i.e., they were significantly up-regulated in some stress conditions, down-regulated in others, and even significantly unaltered upon certain stresses. For example, miR408 was significantly up-regulated in response to cold, drought, HSVd, and *Agrobacterium*, significantly down-regulated in response to short day and *Monosporascus*, and unaltered in response to salinity. However, we detected that a reduced number of miRNAs exhibited a consistent expression pattern. The miR396 and miR6478 expressions were significantly down-regulated in all analyzed situations, except in the case of salinity for miR396 and cold for miR6478 (Fig. 2b and Additional file 6: Figure S4). Although non-significant, lower expression levels relative to control plants were recovered in these latter cases (logFC = − 0.71 in response to LiCl for miR396 and logFC = − 0.32 in response to 20/14 °C for miR6478). A similar situation was observed with the expression patterns of miR168 and miR319, which were down-regulated in all adverse conditions analyzed, but only with statistical significance in four and five conditions, respectively (Additional file 6: Figure S4).

**Fig. 4:**
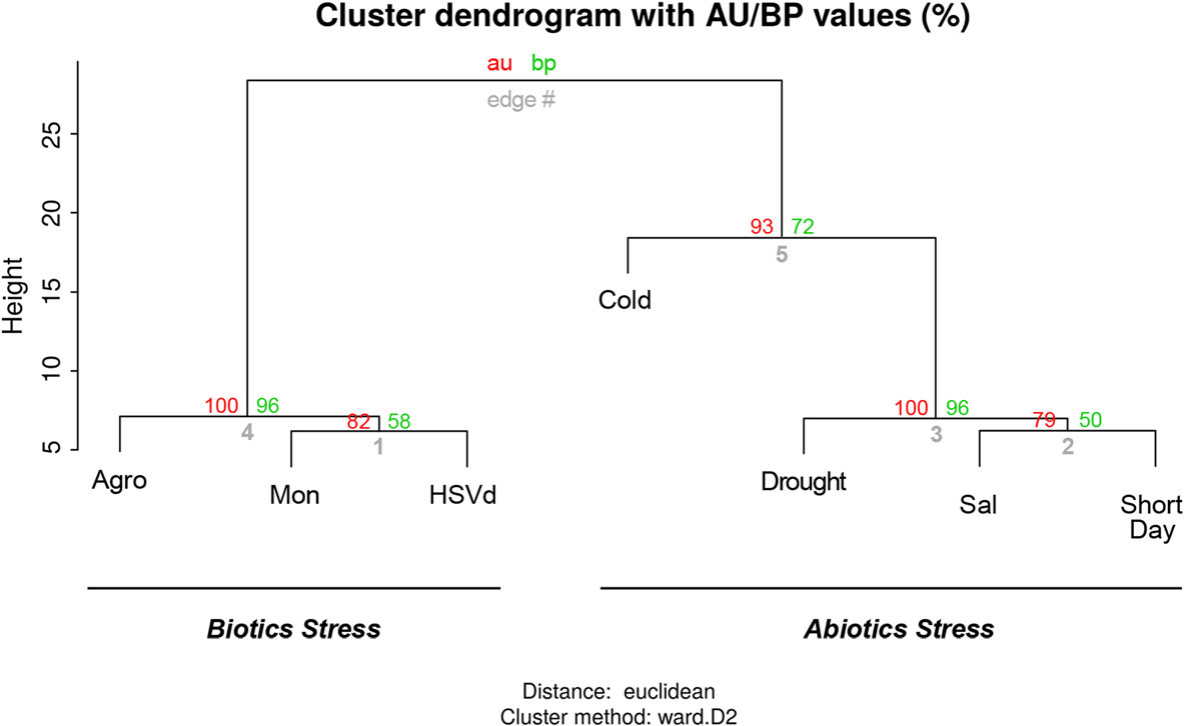
Clustering of stress-responsive miRNAs. Hierarchical cluster analysis of the response to the 7 analyzed stress conditions of the differentially expressed miRNAs according to their total expression values detailed in Additional file 9: Table S3. The values on the edges of the clusters are *P*-values (%). The red values are approximately unbiased (au) *P*-values, and the green values are bootstrap probability (bp) values calculated via multiscale bootstrap resampling (*n* = 100,000)

Interestingly, when the general response to stress was analyzed by clustering of stress-responsive miRNAs according to their total expression values (both differential and non-differential, according to values detailed in Additional file 6: Figure S4), we observed that the miRNA-mediated response to biotic and abiotic stress was significantly distinguished (Fig. 4). Together, these results provide evidence that in melon plants, in agreement with observations in *Arabidopsis* [35], soybean, and rice [36], a high proportion of miRNAs respond to adverse conditions in a stress-dependent manner.

### miRNA-regulated targets in response to stress in melon

To elucidate the information flow mediated by miRNAs in response to stress in melon, we analyzed the existing regulatory relationship between stress-responsive miRNAs and their intended targets. We predicted 22 unique putative targets for the 24 stress-responsive miRNAs (the pairs miR156/157 and miR165/166 share a target) (Additional file 10: Table S4). Consistent with that commonly observed for plant miRNAs [37], putative targets were enriched (13 out of 22) in proteins homologous to classical plant transcription factors (TFs) [SQUAMOSA promoter binding protein-like (SPL), NAC, TEOSINTE BRANCHED/CYCLOIDEA/PROLIFERATIN CELL FACTOR 2 (TCP2), APETALA 2 (AP2), F-BOX, NUCLEAR FACTOR Y (NFY), Scarecrow, ATHB14, etc.], which are associated with development and stress (miR156, miR157, miR159, miR160, miR164, miR165, miR166, miR167, miR169, miR171, miR172, miR319, miR396). This observation is consistent with diverse studies that have shown that miRNA-regulated transcription factors, such as NAC, SPL or TCP play an important role in response to stress in diverse plant species [37, 38]. Other targets were functionally associated with oxidation-reduction processes (miR398, miR408, miR1515, and miR6478), stress response (miR393, miR394, and miR397), RNA silencing (miR168 and miR390), photosynthesis-related processes (miR162), and metal metabolism (miR395). As expected, most of the targets were homologous to genes regulated by the equivalent miRNAs described in diverse plant species.

**Fig. 5:**
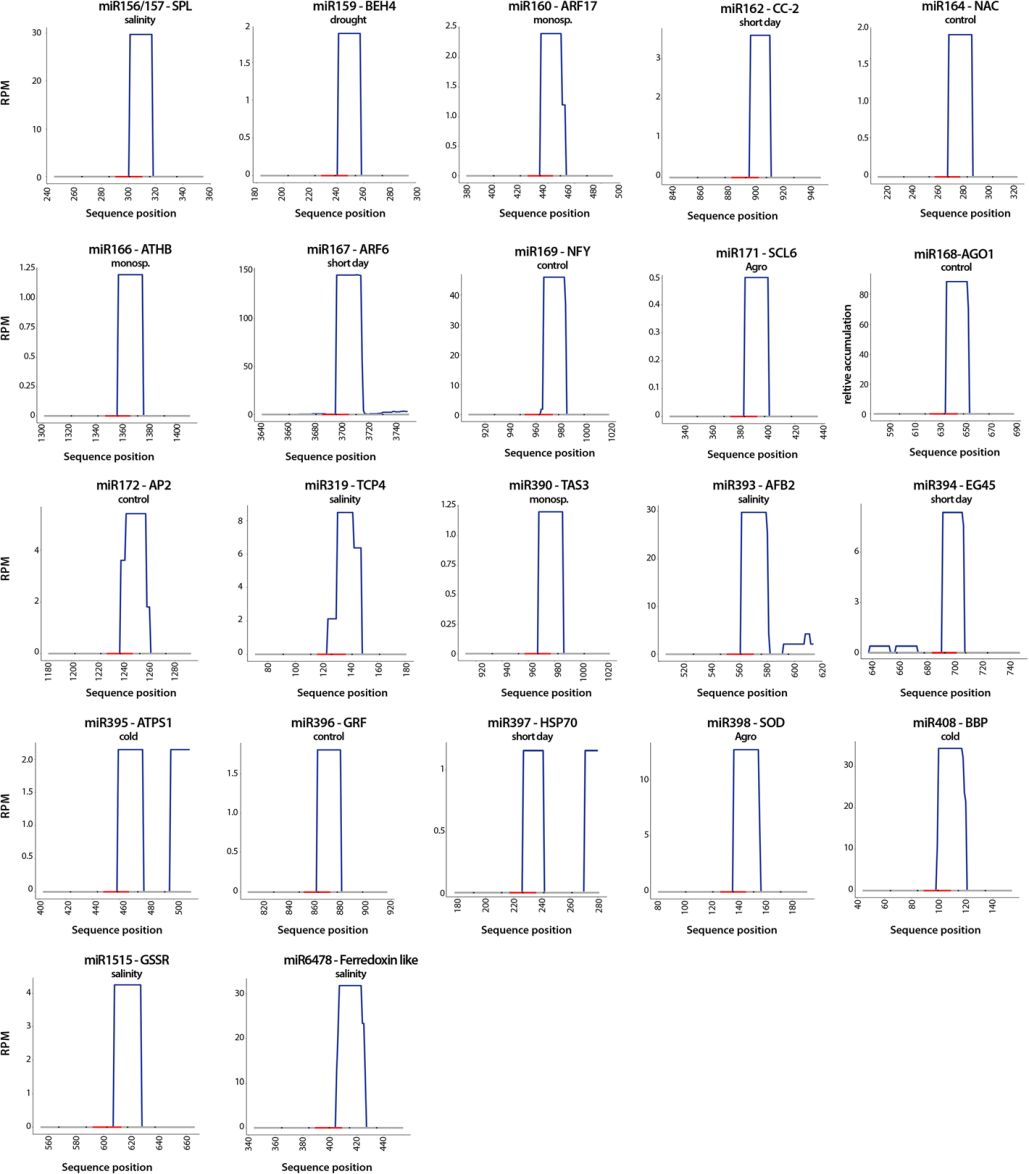
Validation of miRNA predicted targets. Graphic representation of miRNA-cleaved transcripts detected by high-scale degradome assay in representative stress-exposed and control samples. The obtained sequences were plotted (allowing 95% homologous matching) onto the predicted-targets sequences. The red lines on the X-axis indicate the position of the predicted miRNA recognition site in the melon transcripts. The values on the Y-axis represent the number of obtained reads (normalized in reads per million) except for AGO1 transcripts (obtained by conventional 5′-RLM-RACE and represented in percentage of sequenced clones)

To validate the functionality of the predicted miRNA-target interactions, we analyzed by 5′-RLM-RACE the RISC-mediated processing of the 22 transcripts expected to be regulated by stress-responsive melon miRNAs. As shown in Fig. 5, in the entire analyzed melon transcripts, the cleavage positions (between nucleotides 10 and 12 relative to the 5′-end of the miRNAs) estimated by 5′-RLM-RACE in the predicted targets are consistent with those expected for transcripts sliced via miRNA-guided AGO activity [39].

**Fig. 6:**
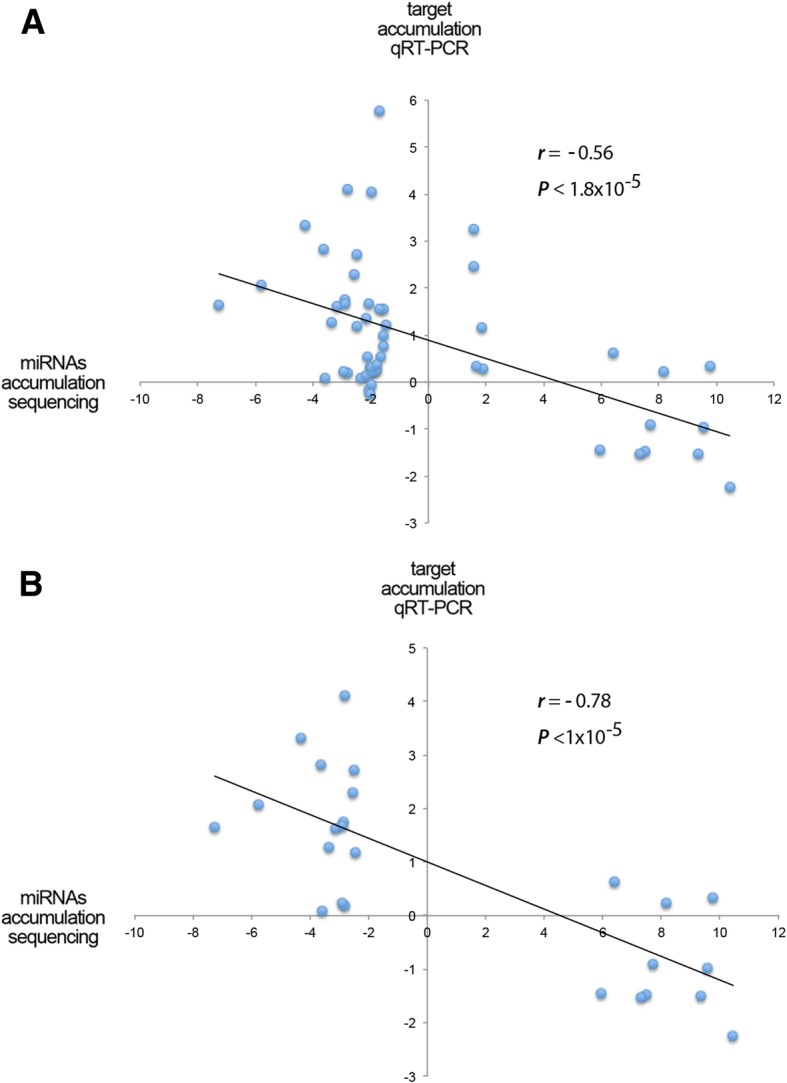
Scatter plot showing the significant negative correlation (estimated by Pearson correlation coefficient) between the expression levels of stress-responsive miRNAs with differential accumulation determined by sequencing [logFC ≤ − 1.5 or ≥ 1.5 in (**a**) or logFC ≤ − 2.5 or ≥ 2.5 in (**b**)] and the accumulation of their predicted targets in the corresponding stress situations estimated by qRT-PCR (detailed information in Additional file 11: Table S5)

To obtain more mechanistic insight about the regulatory role of the melon miRNAs in response to stress, we analyzed the accumulation of protein-coding target transcripts in the stress situations where the responsive miRNAs showed accumulation values with logFC ≤ − 1.5 or ≥ 1.5 (20 out of 22 predicted targets, Additional file 11: Table S5). As expected, a significant negative correlation was obtained when we compared the expression values of stress-responsive miRNAs with the accumulation of their targets estimated by qRT-PCR (Pearson correlation coefficient *r* = − 0.56) (Fig. 6a). The negative correlation was strongly increased (Pearson correlation coefficient *r* = − 0.78) when only miRNAs with differential values with logFC ≤ − 2.5 or ≥ 2.5 were analyzed (Fig. 6b and Additional file 11: Table S5). These results are consistent with the involvement of miRNAs in the fine regulation of the target-transcript stability in response to stress in melon plants.

### Melon miRNAs show broad, intermediate, and narrow response ranges under stress

**Fig. 7:**
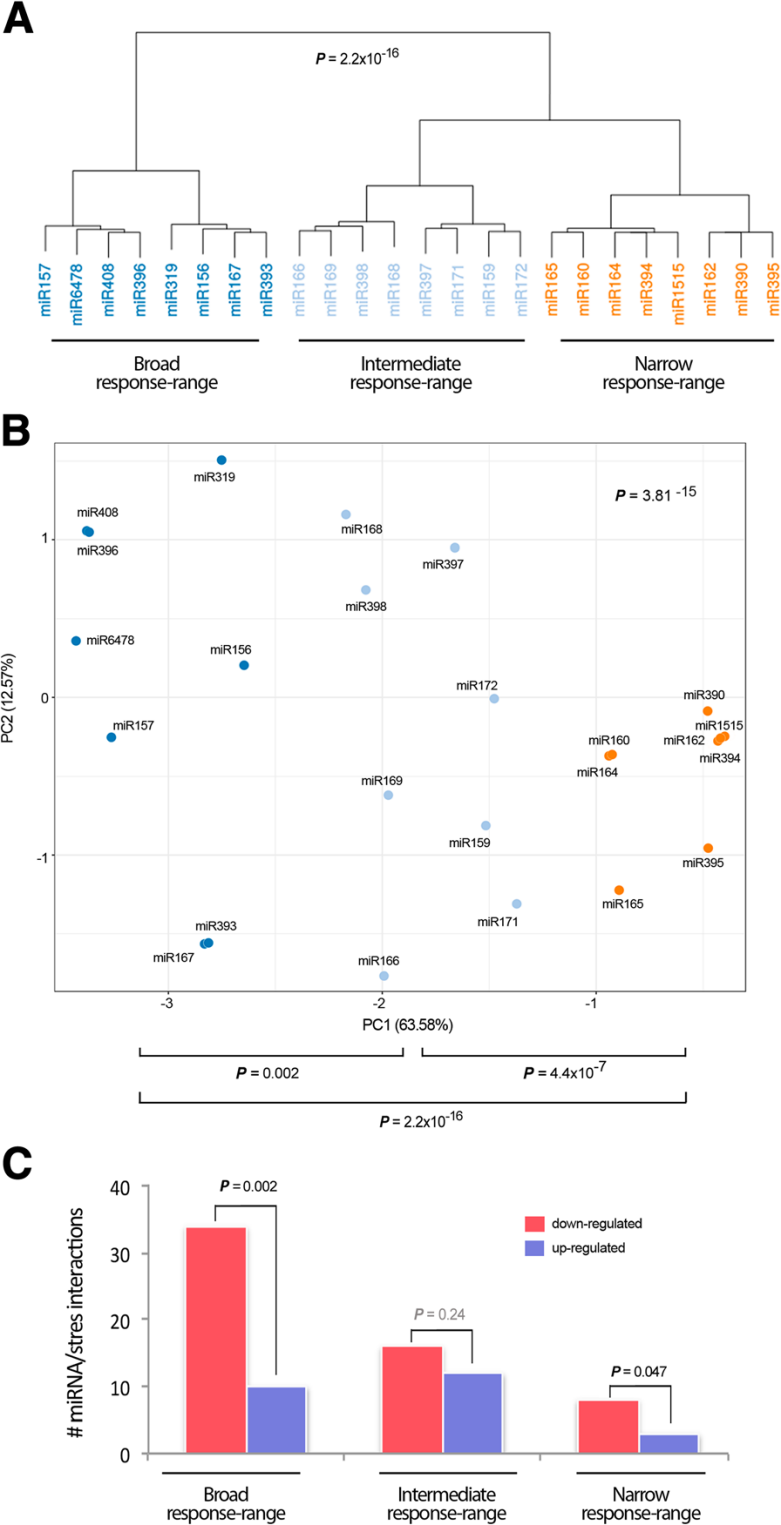
Stress-responsive miRNAs are grouped in relation to its response-range to stress. **a** Dendrogram showing the clustering of stress responsive miRNAs in three main groups according to the analyzed stress conditions. **b** Principal component analysis of stress-responsive miRNAs. In (**a**) and (**b**), the statistical significance of the identified clusters was estimated by Mann-Whitney-Wilcoxon test, considering the inter- and intra-group Euclidean distances. **c**) Graphic representation of the direction (up- or down- regulated) of the melon miRNAs with broad, intermediate, and narrow response range to stress. The statistical significance of the differences was estimated by Mann-Whitney-Wilcoxon test, significant *P*-values are showed in bold (<0.05), and non-significant values in gray

Expression of stress-related miRNAs, as of all transcripts, is expected to be dynamic in response to the type, magnitude, and exposure time of stress. In an attempt to infer the global response of the different miRNAs to the seven environmental conditions tested (referred to the specific exposure times and doses here considered), the 24 stress-responsive miRNAs (determined by differential expression analysis) were organized into a table of presence and absence (Additional file 12: Table S6) in which the values “1” and “0” represent whether or not, respectively, a miRNA is responsive (with either increased or decreased expression) to a stress condition. Correlations between miRNA responses (considering miRNA behavior and the different treatments) were estimated by PCA. Clustering analysis of the obtained values suggested that the miRNA-mediated response to the analyzed conditions might be organized into three different groups, each composed of eight melon miRNAs (Fig. 7a). The statistical significance of the differences between the predicted groups was estimated considering the Euclidean distances between components (cumulative proportion of variance explained by two components of 76.15%) (Fig. 7b). PCA data suggested the existence of a group of melon miRNAs altered in response to a broad range of stress conditions (with modified expression in five or six stresses, either up or down), constituted by miR156, miR157, miR167, miR319, miR393, miR396, and miR408. That is, for instance, miR408 is up-regulated in response to cold, HSVd, drought, and *Agrobacterium*, and down-regulated in response to *Monosporascus* and short day (i.e., only not altered in response to salinity). A second predicted cluster included those miRNAs responsive to an intermediate range (3 or 4) of adverse environmental conditions (miR159, miR166, miR168, miR168, miR171, miR172, miR397, and miR398). Finally, the miRNAs altered (again, either up or down) only under a narrow number (1 or 2) of stress conditions (miR160, miR162, miR164, miR165, miR390, miR394, miR395, and miR1515) were clustered independently. For instance, note that miR395 is only altered (in particular, up-regulated) in response to salinity. These three categories served us to classify miRNAs according to their relevance for the general-purpose concept of stress, but not to link miRNAs to a particular stress condition. Since plant networks are very intricate, it is expected that some miRNA can participate in widely different scenarios, although with different roles.

Next, we attempted to infer the potential relationship between this PCA-predicted organization and the functional response. The analysis of the biological activity, estimated by GO, provided evidence that the targets regulated by the broad response range miRNAs were mainly cell factors related to stress response. Alternatively, the intermediate response range miRNAs were predicted to modulate mainly developmental regulators, and narrow response range miRNAs participated in diverse functions (Additional file 13: Table S7). When the direction of the differential expression (up- or down- regulation) was taken into account, we observed that broad response range miRNAs, as well as those with a narrow response range, showed a significant bias towards down-regulation (Fig. 7c).

### The miRNA-mediated, stress-responsive network in melon

**Fig. 8:**
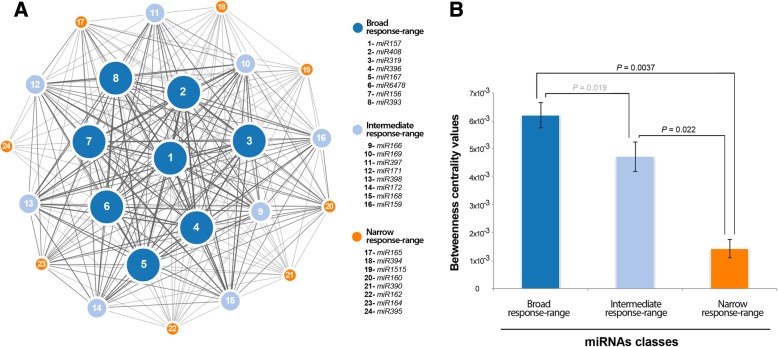
Network of stress-responsive miRNAs in melon. **a** Nodes in the network represent differentially expressed miRNAs. Colors and numbers depict the different groups of stress-responsive miRNAs detected in melon. Node size is proportional to the number of stress conditions where a particular miRNA is differentially expressed (5 or 6 for broad, 3 or 4 for intermediate, and 1 or 2 for narrow response range). Edges represent weighted associations between the terms based on response to common stress conditions. **b** Graphic representation of the average *betweenness* of the nodes calculated for broad, intermediate, and narrow response range miRNAs in melon. The statistical significance of the differences was estimated by Mann-Whitney-Wilcoxon test, significant *P*-values are showed in bold (<0.05), and non-significant values in gray. Error bars represent the standard deviations in *betweenness*

We finally analyzed the coordinated responses of the different miRNAs. For every pair of miRNAs, we searched for stress conditions to which both were responsive (i.e., either up- or down-regulated). When two miRNAs exhibited a response to a particular stress condition, these miRNAs were considered connected. Using this information, summarized in Additional file 12: Table S6, we built a stress-response miRNA network in which nodes represented miRNA families and edges linked miRNAs commonly responding to at least one stress condition. The weight of the edge in the network was normalized such that it represented the strength of the connection between the two miRNAs (the higher the proportion of stress that was common in the response of two miRNAs, the thicker was the edge connecting these miRNAs). Analysis of the miRNA-responsive network in *C. melo* revealed an architecture that was consistent with two main modules (or layers) (Fig. 8a). One central module composed of miRNA families highly connected (broad response range), and another peripheral module comprised of miRNA families with lower connectivity (narrow response range). A more diffuse layer constituted by miRNAs with an intermediate response range was also evident in the network. The statistical robustness of the two different layers in the network was estimated by analysis of the *betweenness* values obtained for each stress-responsive miRNA in the network (Fig. 8b). Betweenness accounts for the degree of centrality of a given node (miRNA in this case) in the network, calculated through the number of shortest paths in which that node is involved [44]. Hence, a miRNA with high betweenness in our network is expected to mediate in multiple stress responses (i.e., broad range).

## Discussion

Since the first plant miRNA was described, considerable growth has been witnessed in the studies aimed at their identification and characterization of biogenesis and functional activity. Increasing evidence reaffirms that miRNAs play fundamental roles in controlling diverse aspects of plant development and plant-environment interactions. Global analyses have demonstrated that diverse miRNAs can modulate the recovery of plant cell homeostasis under external adversities, revealing the existence of another layer of gene regulation (beyond transcription control) in response to stress [13]. Although the value of miRNAs for orchestrating the stress response is relatively well established, there are fundamental questions that remain unanswered: i) what kind of miRNA-based regulatory architectures plants use to respond to environmental stress [1, 12]; ii) how similar or different the miRNA-mediated response is to diverse biotic and abiotic stress situations [7, 13], and iii) to what extent the knowledge gathered in model plants (e.g., Arabidopsis) is suitable to be directly transferred to major agricultural crops [1].

To address, at least in part, these questions, we analyzed known stress-responsive miRNAs in melon plants with synchronized development exposed to diverse biotic and abiotic stress situations. Our results indicate that, in agreement with previous observations in other species, a small group of stress-responsive miRNAs with significant differential accumulation exists in melon plants. We identified 24 known melon miRNA families that showed common or specific altered expression patterns under stress (at least one condition). The entire set of stress-responsive miRNAs identified in melon corresponds to miRNAs previously reported as associated with biotic and abiotic stress in other plants, such as rice, soybean, wheat, cotton, arabidopsis, maize, barley [7, 13, 35, 36, 40, 41], etc. When the behavior of the miRNAs was analyzed in detail, we observed that the direction (up- or down-regulation) of the differential expression was mainly dependent on a given stress, and that only a reduced number of miRNAs were commonly responsive to all stresses. This phenomenon is consistent with the general notion that miRNAs may aid in recovering plant-cell homeostasis in a stress-dependent manner [1].

According to the demand rule of gene regulation [42], the negative mode of regulation (repression) is intended to control the expression of genes whose function is in low demand in the natural environment of the plant (e.g., stress response), whereas the positive mode of regulation (activation) is intended to control the expression of genes whose function is in high demand (e.g., development or metabolism). In this way, we would expect miRNAs to act as key mediators in stress response (i.e., in situations in which the regulation is in low demand), as miRNAs are by definition negative regulators. Note that the stress-response genes could also be repressed transcriptionally. Therefore, we would expect miRNAs to be expressed in normal conditions and be repressed under stress. Notably, this is the general trend that we observed in our data (Fig. 2a). Under the different stress situations analyzed in this work (except for the LiCl treatment), we observed that most of the miRNAs in melon showed significant differential expression in the down direction. The genes regulated by these miRNAs are, in principle, the genes required by the plant to face the stress (Additional file 13: Table S7). They are expected to present any mode of action, i.e., they could go from miRNAs with broad (e.g., miR396) to narrow response range (e.g., miR390). This depends on whether the function controlled by the miRNA is of broad spectrum or not (Additional file 14: Figure S7). However, we also found that in the case of the LiCl treatment (salinity), almost all differently expressed miRNAs are up-regulated. This suggests that, coincident with that observed in other plant species [43], the defence strategy to cope with ionic imbalance, at least the submodule regulated by miRNAs, mainly consists in reducing the metabolic and developmental activities to recover ionic homeostasis and not in expressing new genes for such a task.

Furthermore, a network analysis [44] of the stress-responsive miRNAs in melon was carried out (Fig. 8a). The resulting network linked together all miRNAs with 251 interactions out of a maximum possible of 276 for 24 miRNAs (90.9%). This is an indicator of the intricate regulatory system, in which miRNAs reactive to a broad range of stress conditions are central elements (statistically assessed by means of *betweenness* calculations). Our results, although restricted to the stress conditions analyzed here, suggest that these miRNAs might process a high amount of information within the plant cell and that their expression should be controlled by master signals that trigger in a global manner upon stress irrespective of its nature (e.g., MAP kinase, ROS accumulation, hormones, etc.) [37]. This view is consistent with the observation that broad-range stress-responsive miRNAs identified in melon correspond to miRNAs involved in response to diverse biotic and abiotic stresses in maize, rice, wheat, barley, arabidopsis, and cotton [1, 12]. Alternatively, miRNAs responding narrowly (consequently with lower connectivity) appear in the periphery of the network. This allowed speculation about possible selective stress-related regulatory activity in melon, at least under the type and magnitude analyzed here.

**Fig. 9:**
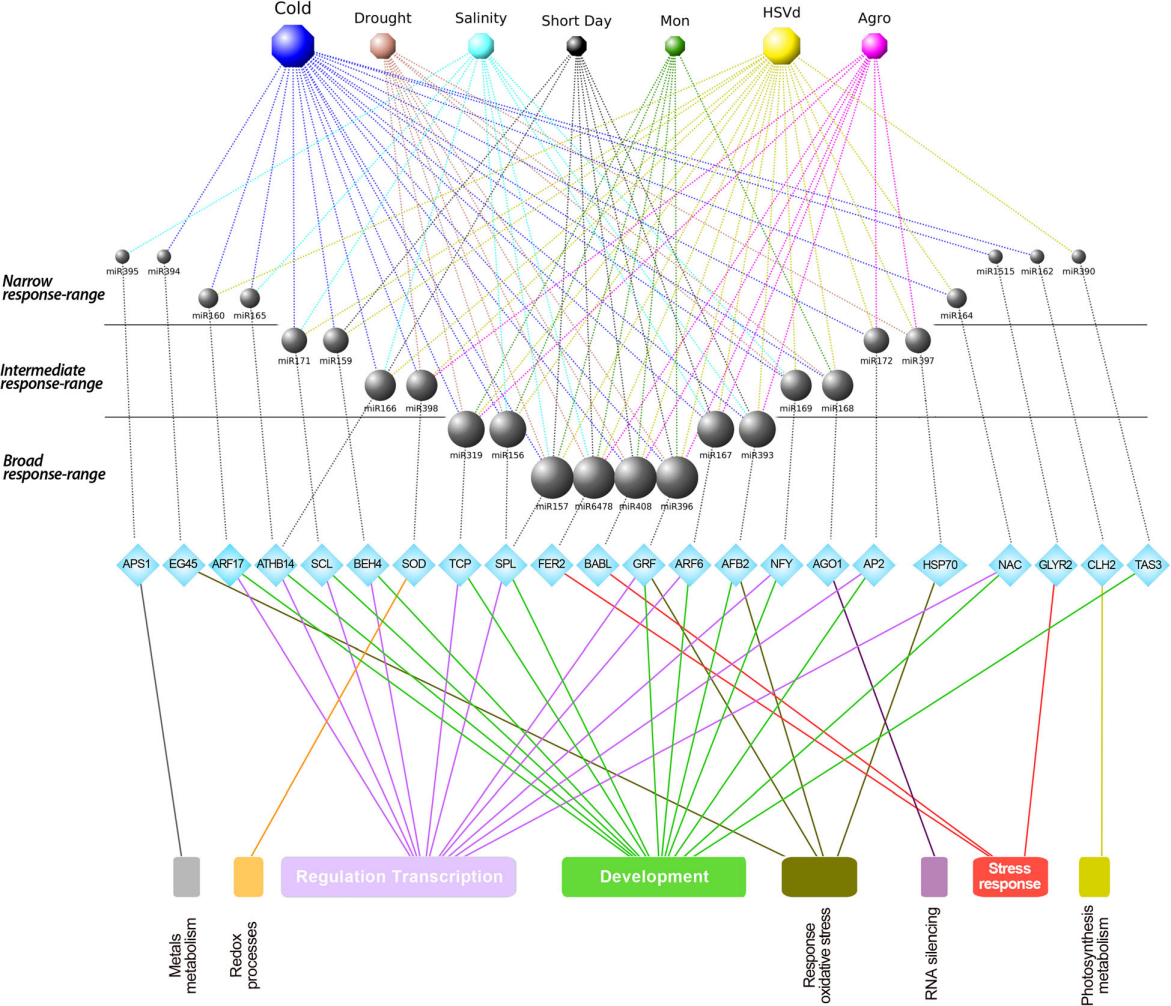
The miRNA-target network involved in stress-response in melon. General overview of the relationship between the stress conditions here analyzed, the differentially expressed miRNAs, and their predicted targets. The biological functions of the targets correspond to the main functional GO terms. Node size for stress conditions, miRNAs, and GO functions is proportional to the number of connections

MiRNAs participate in the response to environmental stress by regulating key components of the complex gene regulatory networks that govern the physiology of the plant [1]. Certainly, it is accepted that many of the stress-responsive miRNAs in plants target TFs [31, 37]. This is in tune with our data, in which many of the transcripts predicted to be regulated by stress-responsive miRNAs were considered putative TFs, homologous to well-known transcription factors in model plants (SPL, NAC, TCP, AP2, F-BOX, NFY, Scarecrow, ATHB14, etc.) (Fig. 9). This suggests that melon, as well as further plant species (arabidopsis, rice, maize, sorghum, sunflower, etc.) [1, 13, 14] exploits miRNAs as modulators of the transcriptional layer to mount an efficient stress response. In this way, a change in miRNA expression can have a large impact on plant physiology through the action of transcription factors (which can be hubs in those gene regulatory networks), working as amplifiers of the response [37, 45]. But the complexity of the picture can be greater and indeed more work is required in this direction to fully unveil the regulatory strategies followed by plants to cope with stress. TFs and miRNAs can be regulating each other establishing feedback and feedforward loops [45].

Importantly, previous works reporting functional associations between miRNAs and TFs under stress support our results in melon. For example, we observed that miR319 down-regulation correlated with high levels of TCP transcripts in melon plants exposed to cold and drought (Additional file 11: Table S5). This resembles the observed pattern in rice and creping bentgrass (*Agrostis stolonifera*), where the regulatory interplay between miR319 and TCP family members was identified to play an important role in the modulation of the plant response to low temperature [46] and water scarcity [36]. Another extensively studied miRNA-mediated circuit regulating stress response in cotton [41], rice [36], and wheat [40] is the one that includes the repression of AP2 by miR172. Degradome assays and transcript accumulation determined by qRT-PCR provided evidence that this regulation can exist in melon exposed to both biotic (*Agrobacterium* and HSVd) and abiotic (cold) stress conditions. Finally, diverse studies have shown that NAC TFs play a fundamental role in plant response to various environmental stresses [1]. Interestingly, it has been suggested that the conserved miR164-targeted NAC genes may modulate development and stress response in rice [47]. These results are consistent with those reported here, in which miR164 down-regulation under cold and HSVd infection correlated with increased accumulation of NAC transcripts. Certainly, further studies (mainly focused on the identification of promoter regions for miRNA transcription and the complete identification of target function) are needed to validate these miRNA-based regulatory circuits and assess to what extent they are central for stress response in melon plants.

In conclusion, the results obtained in this work support the anticipated notion that plants may use miRNA-mediated regulations, highly conserved within the vegetal kingdom, as a pivotal mechanism to quickly respond in adverse environmental situations [1, 37]. Furthermore, this first comprehensive analysis of miRNA expression profiles in response to a variety of biotic and abiotic stresses in a species of agronomical importance (melon) offers the possibility that, in the future, this knowledge may be transferred to breeding programs to obtain new melon varieties with enhanced tolerance to multiple stress conditions.

## Methods

### Plant material, growth conditions, and stress treatments

Melon seeds of cv. *Piel de Sapo* were germinated in Petri dishes at 37 °C/48 h darkness followed by 24 h/25 °C (16/8 light/darkness). We chose this melon cultivar for our research because *Piel de Sapo* it is the predominant biological source for the diverse molecular tools that have been developed for melon in the last years [26–28]. Melon seedlings were sown in pots and maintained for 10 days under controlled conditions **(**28 °C/16 h light and 20 °C/8 h darkness) in a growing chamber. Only plants homogeneously developed were selected for stress treatment. At day 11, selected plants were exposed to the following stress treatments. *i*) *Salinity*: Plants were irrigated with 50 mL of 200 mM LiCl. In this treatment, we used LiCl instead of NaCl to reduce the osmotic effect [48]. *ii*) *Short-day:* Plants were irrigated with 50 mL of Hoagland’s solution and maintained at 28 °C/8 h light and 20 °C/16 h darkness. *iii*) *Cold*: Plants were irrigated with 50 mL of Hoagland’s solution and maintained at 20 °C/16 h light and 14 °C/8 h darkness. *iv*) *Drought*: Plants were irrigated with 50 mL of Hoagland’s solution, then irrigation was suspended until the end of the assay. *v*) *Ascomycete:* Plants were irrigated with 50 mL of Hoagland’s solution plus *Monosporascus cannonballus* mycelium (1000 UFC) grown on potato dextrose agar (PDA) [49]. The spanish isolate used in this work is available in the collection of the Cucurbits Group in the COMAV (https://www.comav.upv.es)*. vi) HSVd:* Plants were irrigated with 50 mL of Hoagland’s solution and inoculated with viroid-RNA (derived from clone JJ3, Y09352**)** in both cotyledons [30]. *vii*) *Agrobacterium:* Plants were irrigated with 50 mL of Hoagland’s solution and infiltrated (without making wounds) in both cotyledons with a suspension of *Agrobacterium tumefaciens* strain C58C1 at 0.8 OD. As a control, we simply used plants irrigated with 50 mL of Hoagland’s solution. Three replicates were performed for each treatment, except in the cases of the control (four) and agrobacterium (two). Each analyzed sample corresponds to a pool of three treated plants. Except for drought treatment, plants were irrigated alternatively (water and Hoagland’s solution) by inundation (1500 mL/48 h). Except for cold and short-day treatments, plants were maintained at 28 °C/16 h light and 20 °C/8 h darkness. At day 11 post-treatment (when the control and treated plants exhibits six true leaves), the first leaf under the apical end per plant was collected in liquid nitrogen and maintained at − 80°C until processing (Additional file 1: Figure S1). This vegetal material was used as source for all analysis performed in this work.

### RNA extraction and small RNA (sRNA) purification

Total RNA was extracted from pooled leaves (~ 0.1 g) recovered from treated and control melon plants using the TRI reagent (SIGMA) according to the manufacturer’s instructions. The low-molecular weight RNA (< 200 nt) fraction was enriched from total RNA using REALTOTAL microRNA Kit (RBMER14, Durviz) according to the manufacturer’s instructions.

### sRNA sequencing

Production and sequencing of the libraries were carried out by SISTEMAS GENOMICOS (https://www.sistemasgenomicos.com). Twenty-four (three biological replicates by each treatment, except for *Agrobacterium* treatment and control, with 2 and 4 replicates respectively) cDNA libraries -individually bar coded- were obtained by following ILLUMINA’s recommendations. Briefly, 3′ and 5′ adaptors were sequentially ligated to the RNA prior to reverse transcription and cDNA generation. cDNAs were enriched by PCR to create the indexed double stranded cDNA library. Size selection was performed using 6% polyacrylamide gel. The quantity of the libraries was determined by quantitative real-time PCR (qRT-PCR) in a LightCycler 480 (ROCHE). Prior to cluster generation in cbot (ILLUMINA), an equimolar pooling of the libraries was performed. The pool of the cDNA libraries was sequenced by paired-end sequencing (100 × 1) in a HiSeq 2000 (ILLUMINA). Adaptors and low quality reads were trimmed by using the Cutadapt tool (v. 1.10) in Python [50].

### Bioinformatic analysis of miRNA sequences

To study the correlation exhibited by the miRNA expression profiles among the different stresses and their biological replicates, principal component analysis (PCA) was used. PCA was performed with the “prcomp” function with scaling in the “stats” R package (v. 3.3.1) [51] Mann-Whitney-Wilcoxon test was performed to assess for significant differences in the data clusters for Euclidean distances calculated between groups and among groups with the “wilcox.test” function in the “stats” R package.

To detect the stress-responsive miRNAs in melon plants, we employed three different statistical testing methods for evaluating differential expression: NOISeq [52], DESeq2 [53], and edgeR [54] R packages for pairwise differential expression analysis of expression data. Differentially expressed sRNAs were filtered using three criteria: *i*) log2-fold change (log2FC) ≥1 or ≤ − 1 for biological significance, *ii*) *P* value < 0.05, and *iii*) base mean ≥ 5, which is the mean of normalized counts of all samples. Small RNAs identified as differentially expressed by the three methods were aligned against the primary microRNA sequence repository, miRBase (release 21) [32], using blastall (v. 2.2.17). Fully homologous miRNAs to previously described mature melon miRNAs, together with the reads 100% homologous to known *Viridiplantae* miRNAs [with precursor sequence on hairpin structure predicted by miRCat pipeline (UEA sRNA workbench v. 25.0, plant version) in the *C. melo* genome] were identified as known stress-responsive miRNAs.

To determine the general sense of the expression for each miRNA family we employed the median value of expression estimated by box-plot analysis of all family-related sequences under each stress condition considering the log2FC values obtained by DESeq2 analysis (Additional file 7: Figure S5). These values were used to generate Heat maps using the *gplots* R package (v. 3.0.1).

### qRT-PCR assays

Quantification of twelve selected miRNAs was performed starting from low-molecular weight RNA (<200 nt) fractions obtained as described above. Stem-loop-specific reverse transcription for miRNAs detection was performed as previously described [55] using a RevertAid cDNA Synthesis Kit (Thermo Scientific). qRT-PCR assays were performed using PyroTaq EvaGreen mix Plus (ROX) (CulteK Molecular Bioline) according to the manufacturer’s instructions. To analyze target expression, total RNA (1.5 μg) extracted from control or treated plants was subjected to DNase treatment (EN0525, Thermo Scientific™) followed by reverse transcription using RevertAid First Strand cDNA Synthesis Kit (Thermo Scientific™) according to the manufacturer’s instructions for use with oligo-dT. cDNAs were amplified by conventional end point RT-PCR using specific primers (Additional file 15: Table S8) to assess for sequence specificity. Then, real-time PCR was performed as described above. All analyses were performed in triplicate on an ABI 7500 Fast-Real Time qPCR instrument (Applied Biosystems) using a standard protocol. The efficiency of PCR amplification was derived from a standard curve generated by four 5-fold serial dilution points of cDNA mixed from the two samples. RNA expression was quantified by the comparative ΔCt method. Primers used are listed in Additional file 16: Table S9.

### Degradome assay

Degradome libraries were constructed following the protocol previously described [56] with minor modifications. In brief, 225 ng of poly (A+) RNA purified from total RNA using the Oligotex mRNA kit (Qiagen) was used as starting material. Then, a 5’ RNA oligonucleotide adaptor (PARE 5′ Adaptor) containing a MmeI recognition site was ligated to the 5′-phosphate of the truncated poly (A+) RNA by T4 RNA ligase. The ligated products were purified by ethanol precipitation and subjected to a reverse transcription reaction with an oligo dT primer with a known tail (dT primer). Following RNA degradation by alkaline lysis, the purified cDNA was amplified by PCR using adaptor and oligo dT-specific primers (RP1S and GR3’, respectively), digested with *Mme* I and ligated to a 3′ double DNA adaptor (dsDNA-top and dsDNA-bottom). The ligated products were run on a polyacrylamide gel, and those with the expected size were gel-purified and amplified with 30 PCR cycles to finally fill out the sequence of the Illumina 5′ (RP1M) and 3′ adaptors (RPIndex). PCR products of the expected size were gel-purified and subjected to sequencing by Illumina technology. Primers used are listed in Additional file 17: Table S10 A.

The determination of the microRNA-directed cleavage site of AGO1 transcripts (non identified by high-scale degradome), was performed by 5′-RLM-RACE (3739) according to the GeneRacer kit guide (Invitrogen, Carlsbad, CA). Primers used are listed in Additional file 17: Table S10 B. Final PCR products were cloned and five clones were sequenced, allowing determination of mRNA cleavage sites.

### Hierarchical clustering

The general response to the seven analyzed stress conditions of the differentially expressed miRNAs was estimated from the data contained in Additional file 5: Figure S3. The *pvclust* function R-package was used to compute a hierarchical clustering via multiscale bootstrap resampling (*n* = 100.000). For identification of broad, intermediate and narrow response range miRNAs, the 24 stress-responsive miRNAs (determined by differential expression analysis) were organized in a binary table of presence and absence (Additional file 12: Table S6), in which the values “1” and “0” represent whether or not, respectively, a miRNA is responsive (with both either increased or decreased expression) to a stress condition. The *hclust* function R-package was used to compute a hierarchical clustering. The statistical significant was estimated with a Mann-Whitney-Wilcoxon test. Both analyses were performed with Euclidean distance metric and Ward linkage with the “ward.D2” algorithm.

### Analysis of miRNA-mediated networks

The network represents the relationship among stress-responsive miRNAs and common stress conditions. The relationship among the miRNAs were established pairing miRNAs and the common stresses they share, the nodes of the network would correspond to the miRNAs and the edges to the common stresses between both miRNAs. To build the network, two input tables were needed: one, containing information about the nodes and the other about the edges. The nodes table (Additional file 18: Table S11), contains three columns: i) name of the stress-responsive miRNA, ii) group to which it belongs (broad, intermediate, and narrow response range) and iii) number of stresses in which it is present. The edges table (Additional file 19: Table S12), also contains three columns: i and ii) each miRNA in the pair and iii) number of common stresses for each miRNA pair.

The network was visualized with the d3Network package from R [57], creating a D3 JavaScript network useful for studying complex networks and integrating them with any type of attribute data, such that nodes represent families of responsive miRNAs and edges link miRNAs that respond to at least one common stress. To account for the strength of the link between two miRNAs (i.e., the number of stresses to which they both respond), we made the thickness of the edge proportional to the number of stresses in common between linked miRNAs with the thickness of the edge being greater for miRNAs sharing more stresses (divided by the number of total stresses). The size of the nodes is a visual representation of those considered as broad, intermediate, and narrow response range miRNAs.

With the objective to measure the centrality and influence of a miRNA or a group of miRNAs in a common context, the *igraph* package from R [58] was used to calculate the *betweenness* centrality for all nodes of the network. *Betweenness* comes down to counting how many shortest paths go through a given node. The statistical significance of the differences between the different groups (broad, intermediate, and narrow response range) was estimated with a Mann-Whitney-Wilcoxon test.

## Additional files

Additional file 1: **Figure S1.** A) Graphic representation of the experimental approach used in this work to identify by high-throughput sequencing stress-responsive miRNAs in melon plants exposed to 7 (cold, drought, salinity, short day, Monosporascus, HSVd and Agrobacterium) adverse environmental conditions. Non-treated melon plants were used as control. In total 24 independent libraries (three replicates by treatmen except for control − 4- and Agrobacterium − 2-) were analysed. Differentially expressed miRNAs were filtered using three criteria: i) log2 fold change (log2FC) ≥1 or ≤ − 1 for biological significance, ii) *P* value < 0.05, and iii) base mean ≥ 5. Only the sequences predicted as differentially accumulated by the three analysis tools were considered as true stress-responsive miRNAs. B) Detail of stress treatments used in this work. (TIF 39650 kb)

Additional file 2: **Figure S2.** A, Diagram showing the mens of the accumulation of rRNA, tRNA, snoRNA and snRNA derived sRNAs in all analysed samples. Error bars show the confidence interval of the difference between means. B, Diagram showing the accumulation and distribution of the unique clean reads of melon sRNAs ranging between 20 and 25 nt obtained from sequenced libraries. The control and the different analyzed treatments are represented with colors. (TIF 32568 kb)

Additional file 3: **Table S1.** Detail of reads number and size distribution of melon sRNAs sequenced in this work. (TIF 15087 kb)

Additional file 4: **Table S2.** Detail of the sRNAs sequences belonging to know miRNAs families identified as stress responsive in melón. (PDF 1429 kb)

Additional file 5: **Figure S3.** Graphic representation of the expression values (estimated by edge-R) of sRNA sequences recovered from melon exposed to different stress conditions. The dots indicate the expression value of each sRNA. Dotted red lines show the threshold values for differential expression Log2FC > 1.0 or < − 1.0. The dot colors represent significant differentially expressed sequences identified as sRNAs (red) or miRNAs identical to previously described miRNAs in the miRBase (green). (TIF 28419 kb)

Additional file 6: **Figure S4.** Detailed Analysis of expression levels of stress-responsive miRNAs. Heat map showing the expression values (significant and non-significant) obtained for each miRNA family in response to analyzed stress conditions. The dendrogram represent the clustering of miRNAs according to their expression values. (TIF 34307 kb)

Additional file 7: **Figure S5.** To determine the general sense of the expression for each miRNA family we employed the median value of expression (represented by internal box-line) estimated by box-plot analysis of all family-related sequences (red dots). The differential expression values represented in the figure correspond to the log2FC obtained by DESeq2 analysis. (TIF 24420 kb)

Additional file 8: **Figure S6.** Relative accumulation (expressed as Log2FCΔΔCT) with respect to untreated control (Ctrl.), estimated by stem loop qRT-PCR, of 12 representatives miRNAs in melon plants exposed to the seven stress conditions analyzed in this work. C: cold, D: drought, S: salinity, SD: short Day, M: monosporascus, H: HSVd and A: agrobacterium. Error bars show the confidence interval of the difference between means. Only Log2FC values > 1 or < − 1 were considered as indicative of significant differential expression. (TIF 8929 kb)

Additional file 9: **Table S3.** Detailed information of the accumulation values of representative stress-responsive and non stress-responsive melon miRNAs established by sequencing and additionally validated by stem loop qRT-PCR. The values are in Log2 scale. NS: non sequence data (sRNA reads below the limits established to be included in the differential analysis, see materials and methods). (TIF 23836 kb)

Additional file 10: **Table S4.** Description and detailed information of predicted targets for stress-responsive miRNAs identified en melon plants. The GO terms were estimated in base to information for homologous transcripts in arabidopsis. The putative non protein-coding TAS3 predicted as target for miR390 has not GO terms. (TIF 19740 kb)

Additional file 11: **Table S5.** Detailed information of the values used for calculate the correlation between stress-responsive miRNAs expression values -estimated by sequencing- and targets accumulation -estimated by qRT-PCR-. The values are in Log2 scale. In gray are marked the miRNA/target combinations represented in the Fig. 6b. (PDF 229 kb)

Additional file 12: **Table S6.** Table of presence and absence of stress-responsive miRNAs in melon plants. The values “1” and “0” represent respectively if whether or not a miRNA is responsive (with both either increased or decreased expression) to a specific stress condition. 1: stress-responsive, 0: non stress-responsive. (TIF 10502 kb)

Additional file 13: **Table S7.** Functional relation between broad-, intermediate- and narrow-range stress-responsive miRNAs and their predicted targets in melon plants. Main functional GO terms of the targets are also detailed. (TIF 5732 kb)

Additional file 14: **Figure S7.** Possible modes of miRNA-guided target gene regulation under stress in melon. miRNAs that are positively regulated by stress might target genes involved in normal plant functioning. By contrast, broad- and narrow response-range miRNAs that are significantly down-regulated during analyzed stresses are expected to target essential genes ligated to stress-response. This regulatory circuit may exist in two predominant (but not exclusive) forms, I) single: (dotted box) where miRNAs regulate targets mainly associated to specific stress response, and II) double layered: (filled box) where generalist miRNA predominantly target cell factors (SRF) that positively regulate diverse genes involved in stress tolerance and associated to response to multiple stress conditions. (TIF 15544 kb)

Additional file 15: **Table S8**. Detail of the primers used for stem-loop qRT-PCR analysis of melon miRNAs. (TIF 14782 kb)

Additional file 16: **Table S9.** Detail of the primers used for qRT-PCR analysis of transcripts targets of stress-responsive miRNAs. (TIF 9127 kb)

Additional file 17: **Table S10.** Oligos used. (PDF 37 kb)

Additional file 18: **Table S11.** Nodes input table. Column i) indicate the name of the stress-responsive miRNA, ii) group to which they belong and iii) number of stresses in which they are present. 10: represents miRNAs responsive to 5 and 6 stress conditions, 6: represents miRNAs responsive to 3 and 4 stress conditions, and 4: represents miRNAs responsive to 1 and 2 stress conditions. (TIF 12081 kb)

Additional file 19: **Table S12.** Edge input table. Values in source and target columns refers to position of the each stress responsive miRNAs in the nodes table. (PDF 25 kb)

## Abbreviations

AGO1: ARGONAUTE 1
AP2: APETALA 2
DCL1: DICER-LIKE 1
HSVd: Hop stunt viroid
miRNA: microRNA
NFY: NUCLEAR FACTOR Y
PCA: Principal component analysis
RISC: RNA-induced silencing complex
SPL: SQUAMOSA promoter binding protein-like
TCP: TEOSINTE BRANCHED/CYCLOIDEA/PROLIFERATIN CELL FACTOR
TF: Transcription factor
