# Supplementary figures and images for: Inferring the regulatory network of the miRNA-mediated response to biotic and abiotic stress in melon
Source: BMC Plant Biol. 2019 Feb 18;19:78. doi: 10.1186/s12870-019-1679-0 (PMC6379984; doi:10.1186/s12870-019-1679-0)

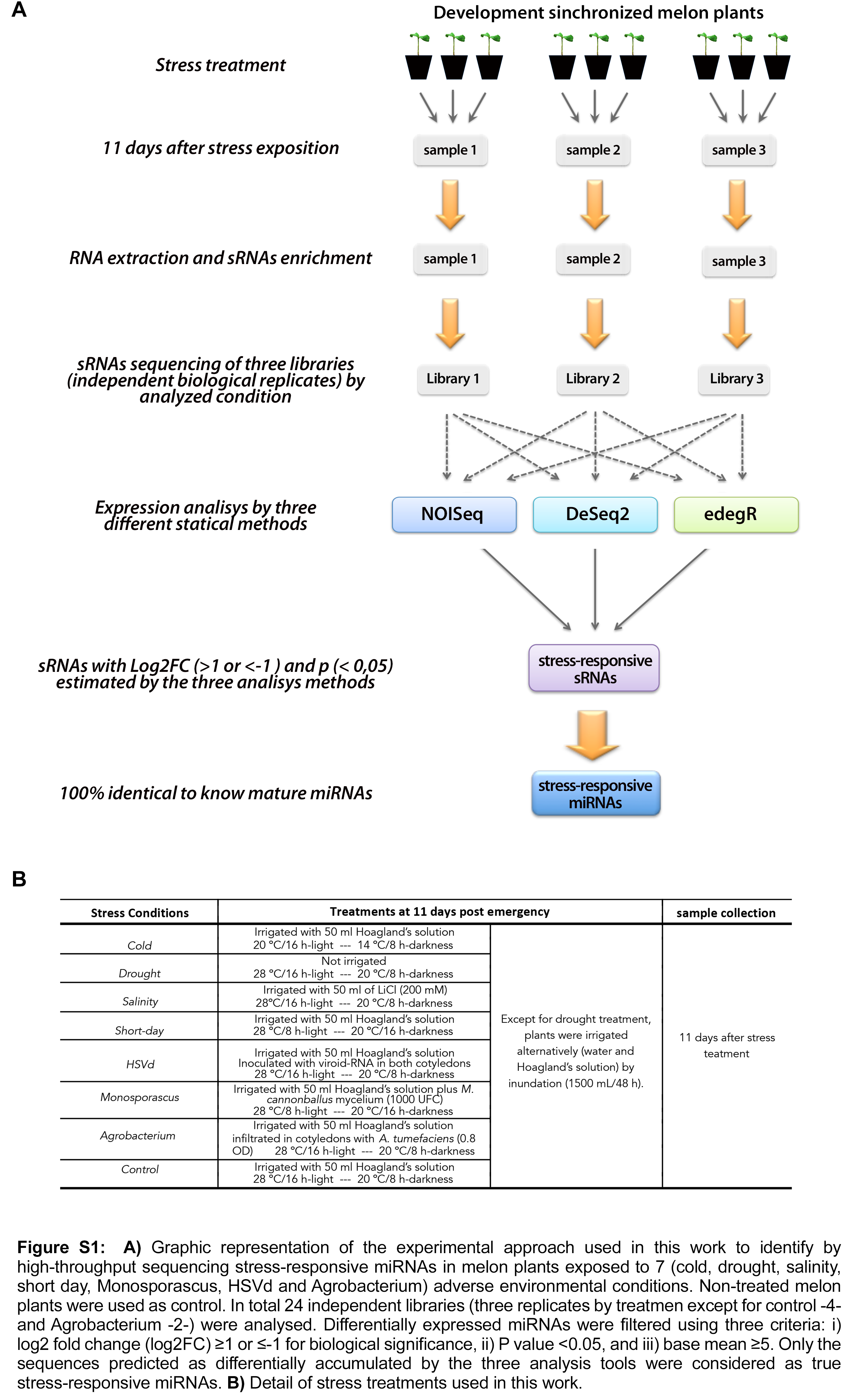

Supplement: Supplementary file 1 — Figure S1. A) Graphic representation of the experimental approach used in this work to identify by high-throughput sequencing stress-responsive miRNAs in melon plants exposed to 7 (cold, drought, salinity, short day, Monosporascus, HSVd and Agrobacterium) adverse environmental conditions. Non-treated melon plants were used as control. In total 24 independent libraries (three replicates by treatmen except for control − 4- and Agrobacterium − 2-) were analysed. Differentially expressed miRNAs were filtered using three criteria: i) log2 fold change (log2FC) ≥1 or ≤ − 1 for biological significance, ii) P value < 0.05, and iii) base mean ≥ 5. Only the sequences predicted as differentially accumulated by the three analysis tools were considered as true stress-responsive miRNAs. B) Detail of stress treatments used in this work. (TIF 39650 kb) [file 12870_2019_1679_MOESM1_ESM.tif]

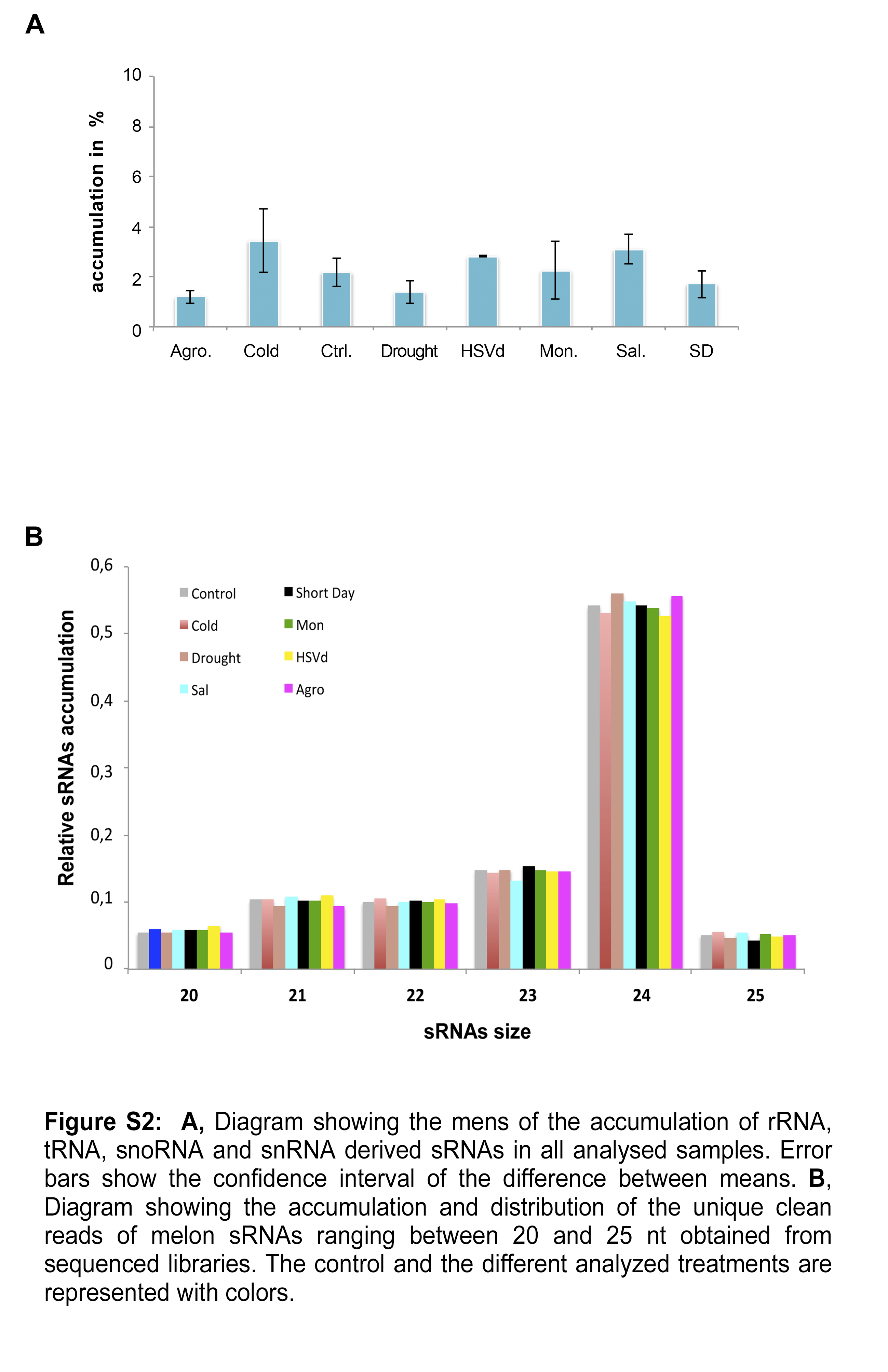

Supplement: Supplementary file 2 — Figure S2. A, Diagram showing the mens of the accumulation of rRNA, tRNA, snoRNA and snRNA derived sRNAs in all analysed samples. Error bars show the confidence interval of the difference between means. B, Diagram showing the accumulation and distribution of the unique clean reads of melon sRNAs ranging between 20 and 25 nt obtained from sequenced libraries. The control and the different analyzed treatments are represented with colors. (TIF 32568 kb) [file 12870_2019_1679_MOESM2_ESM.tif]

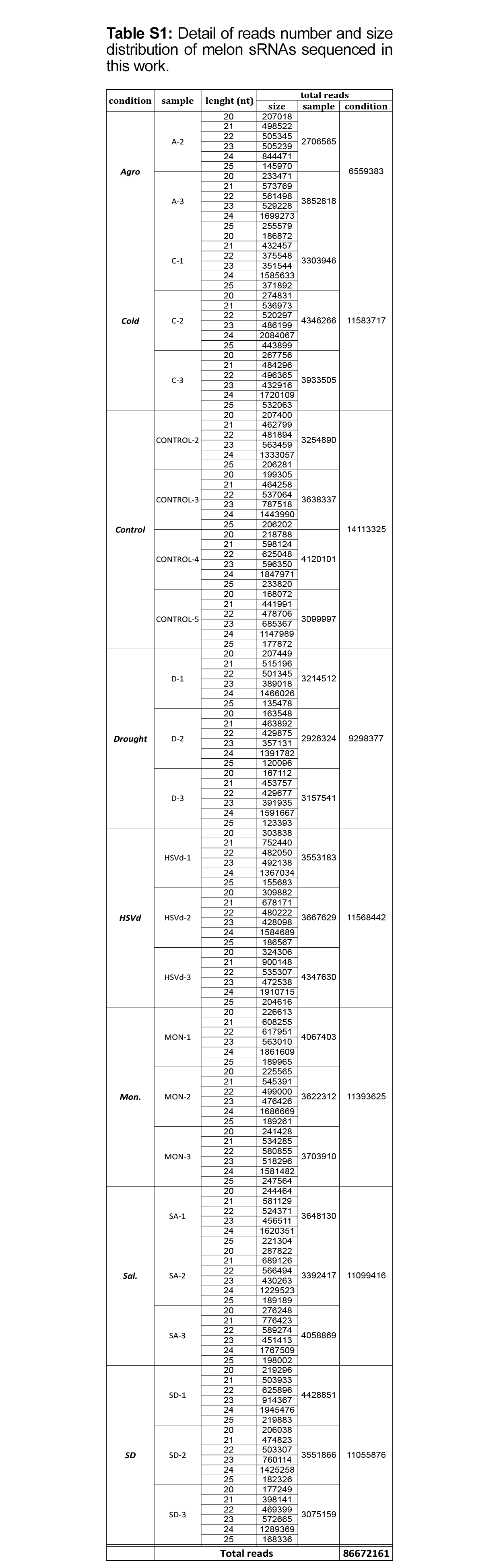

Supplement: Supplementary file 3 — Table S1. Detail of reads number and size distribution of melon sRNAs sequenced in this work. (TIF 15087 kb) [file 12870_2019_1679_MOESM3_ESM.tif]

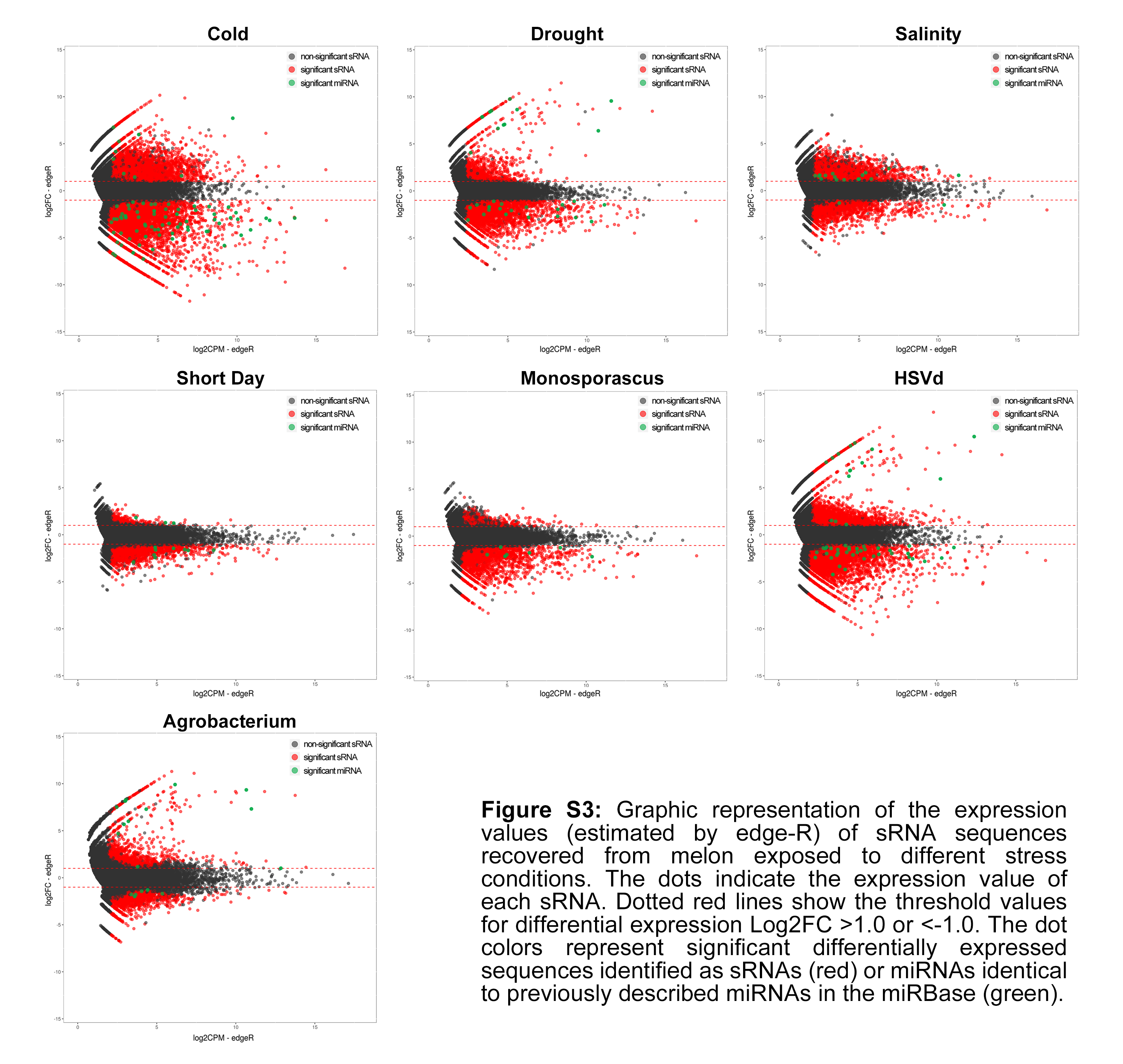

Supplement: Supplementary file 5 — Figure S3. Graphic representation of the expression values (estimated by edge-R) of sRNA sequences recovered from melon exposed to different stress conditions. The dots indicate the expression value of each sRNA. Dotted red lines show the threshold values for differential expression Log2FC > 1.0 or < − 1.0. The dot colors represent significant differentially expressed sequences identified as sRNAs (red) or miRNAs identical to previously described miRNAs in the miRBase (green). (TIF 28419 kb) [file 12870_2019_1679_MOESM5_ESM.tif]

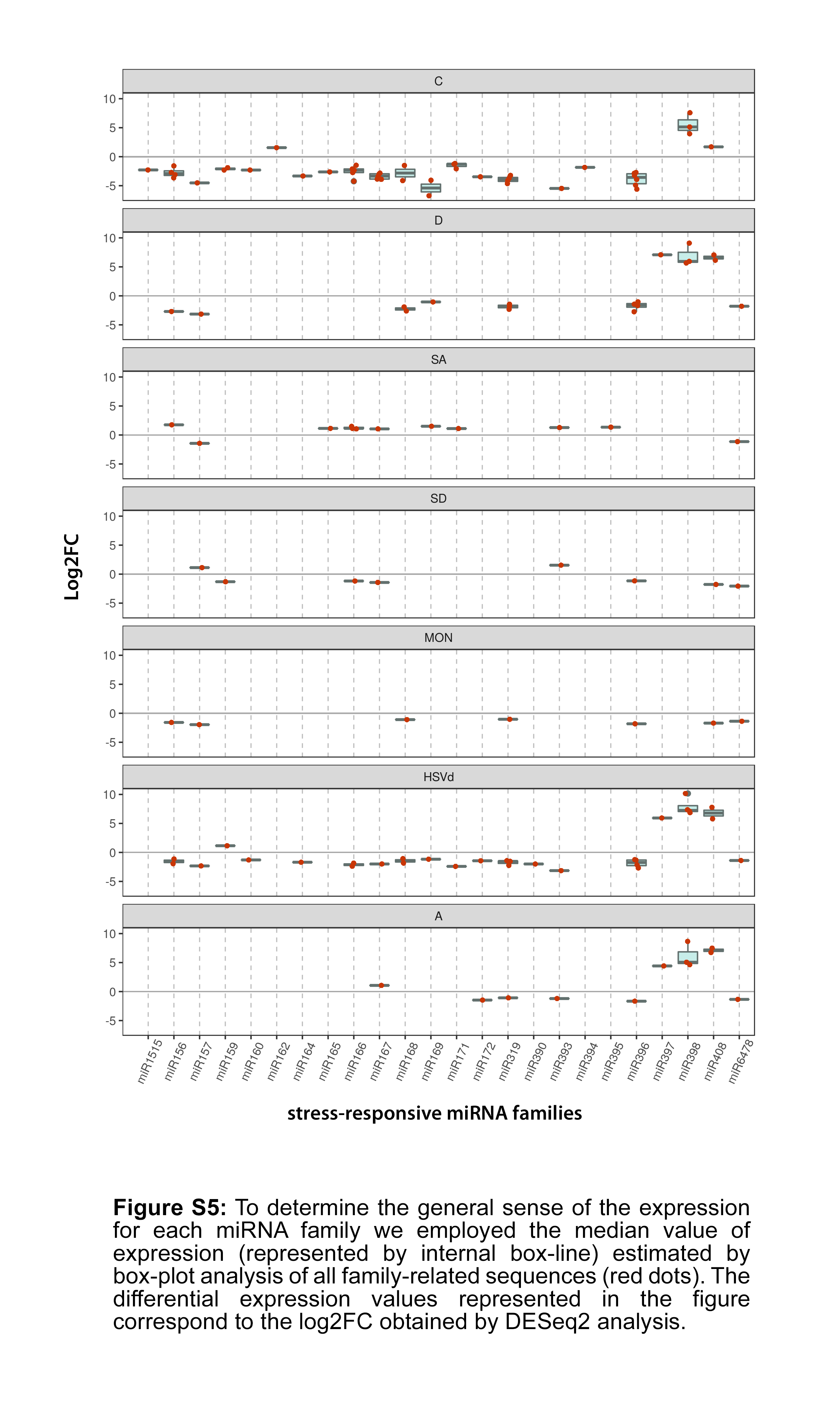

Supplement: Supplementary file 6 — Figure S4. Detailed Analysis of expression levels of stress-responsive miRNAs. Heat map showing the expression values (significant and non-significant) obtained for each miRNA family in response to analyzed stress conditions. The dendrogram represent the clustering of miRNAs according to their expression values. (TIF 34307 kb) [file 12870_2019_1679_MOESM7_ESM.tif]

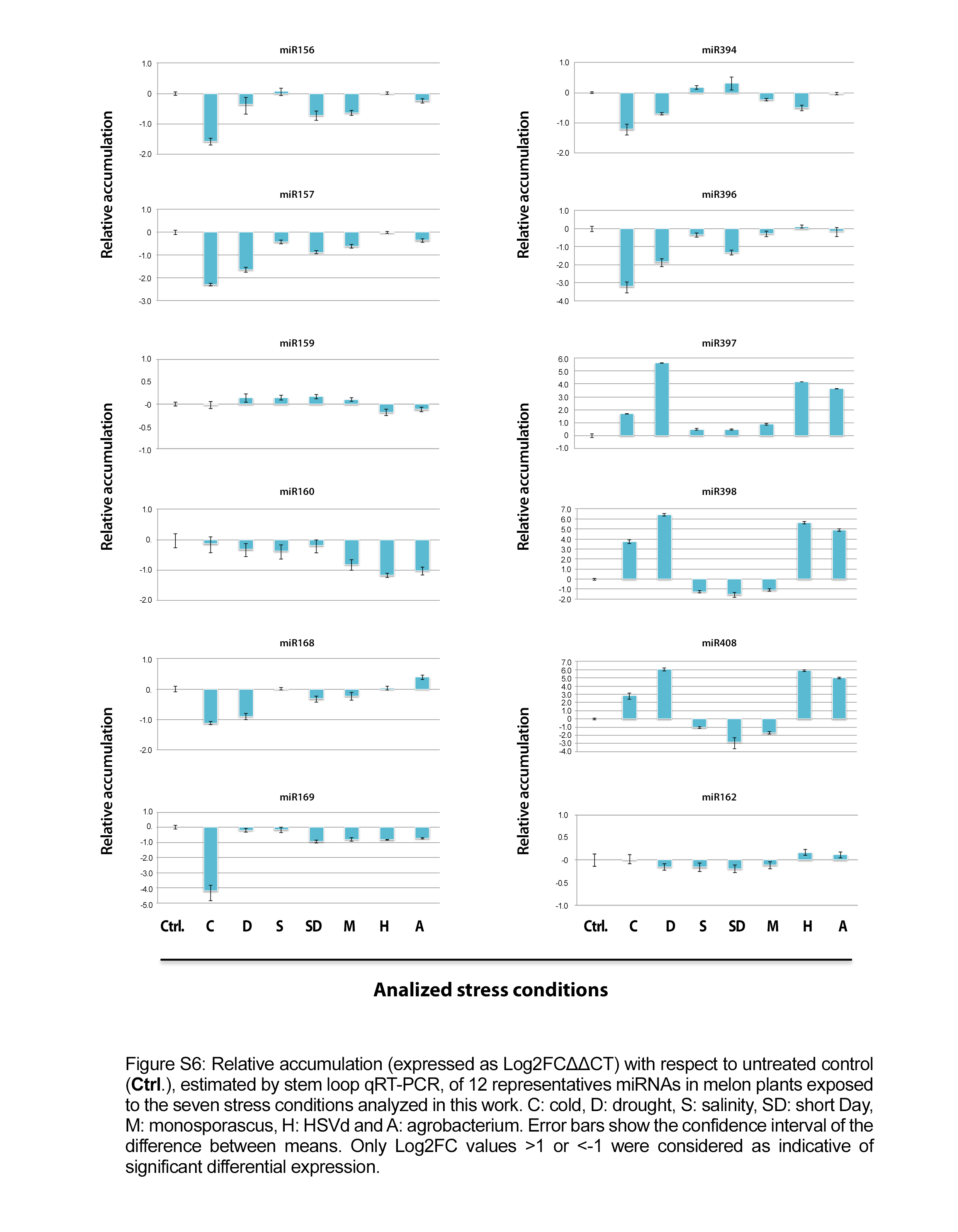

Supplement: Supplementary file 7 — Figure S5. To determine the general sense of the expression for each miRNA family we employed the median value of expression (represented by internal box-line) estimated by box-plot analysis of all family-related sequences (red dots). The differential expression values represented in the figure correspond to the log2FC obtained by DESeq2 analysis. (TIF 24420 kb) [file 12870_2019_1679_MOESM8_ESM.tif]

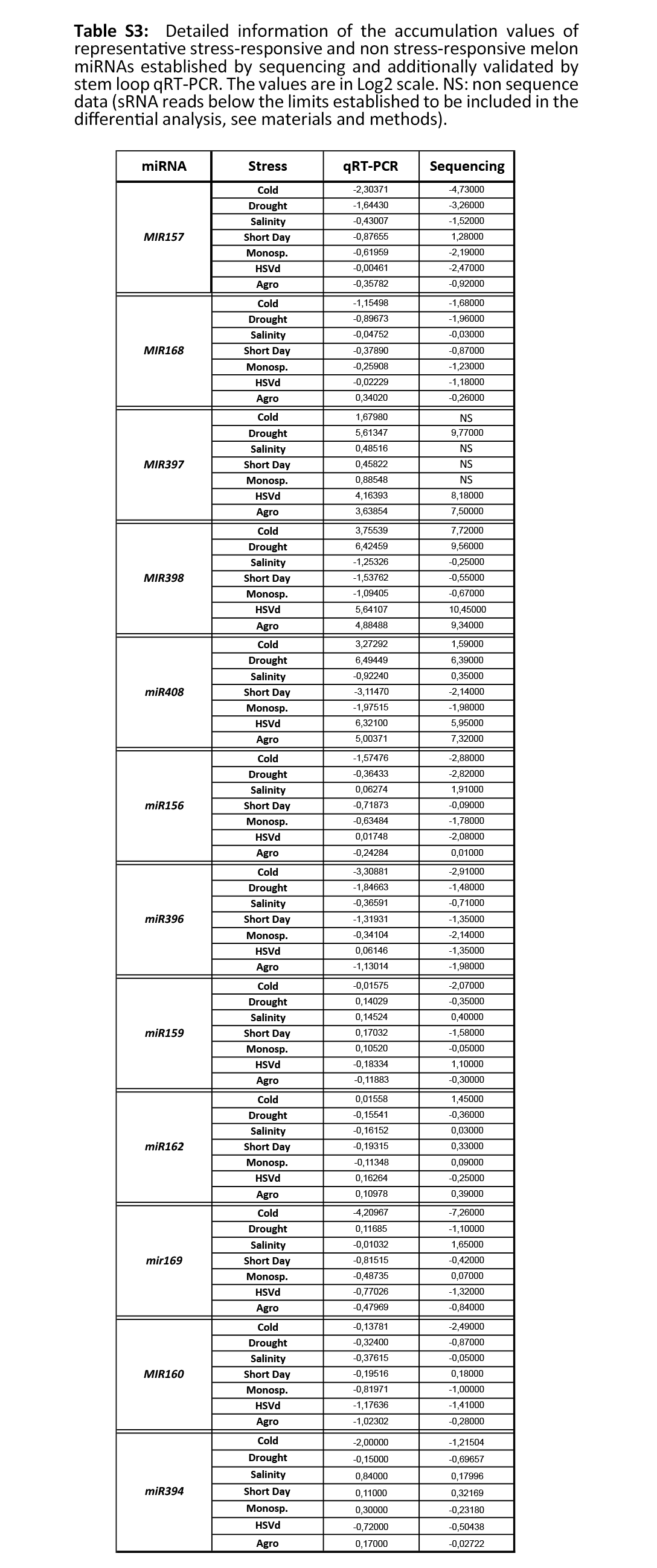

Supplement: Supplementary file 8 — Figure S6. Relative accumulation (expressed as Log2FCΔΔCT) with respect to untreated control (Ctrl.), estimated by stem loop qRT-PCR, of 12 representatives miRNAs in melon plants exposed to the seven stress conditions analyzed in this work. C: cold, D: drought, S: salinity, SD: short Day, M: monosporascus, H: HSVd and A: agrobacterium. Error bars show the confidence interval of the difference between means. Only Log2FC values > 1 or < − 1 were considered as indicative of significant differential expression. (TIF 8929 kb) [file 12870_2019_1679_MOESM9_ESM.tif]

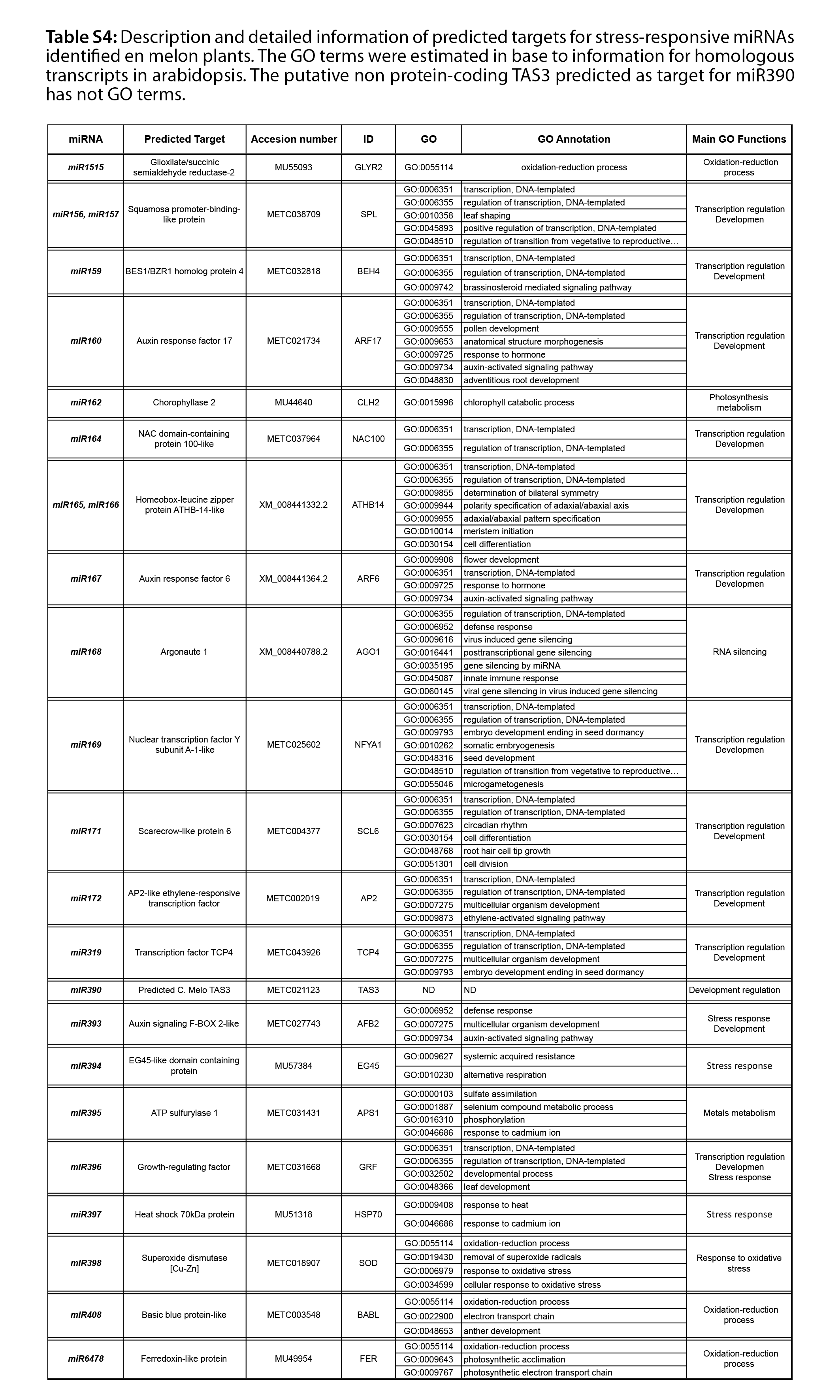

Supplement: Supplementary file 9 — Table S3. Detailed information of the accumulation values of representative stress-responsive and non stress-responsive melon miRNAs established by sequencing and additionally validated by stem loop qRT-PCR. The values are in Log2 scale. NS: non sequence data (sRNA reads below the limits established to be included in the differential analysis, see materials and methods). (TIF 23836 kb) [file 12870_2019_1679_MOESM10_ESM.tif]

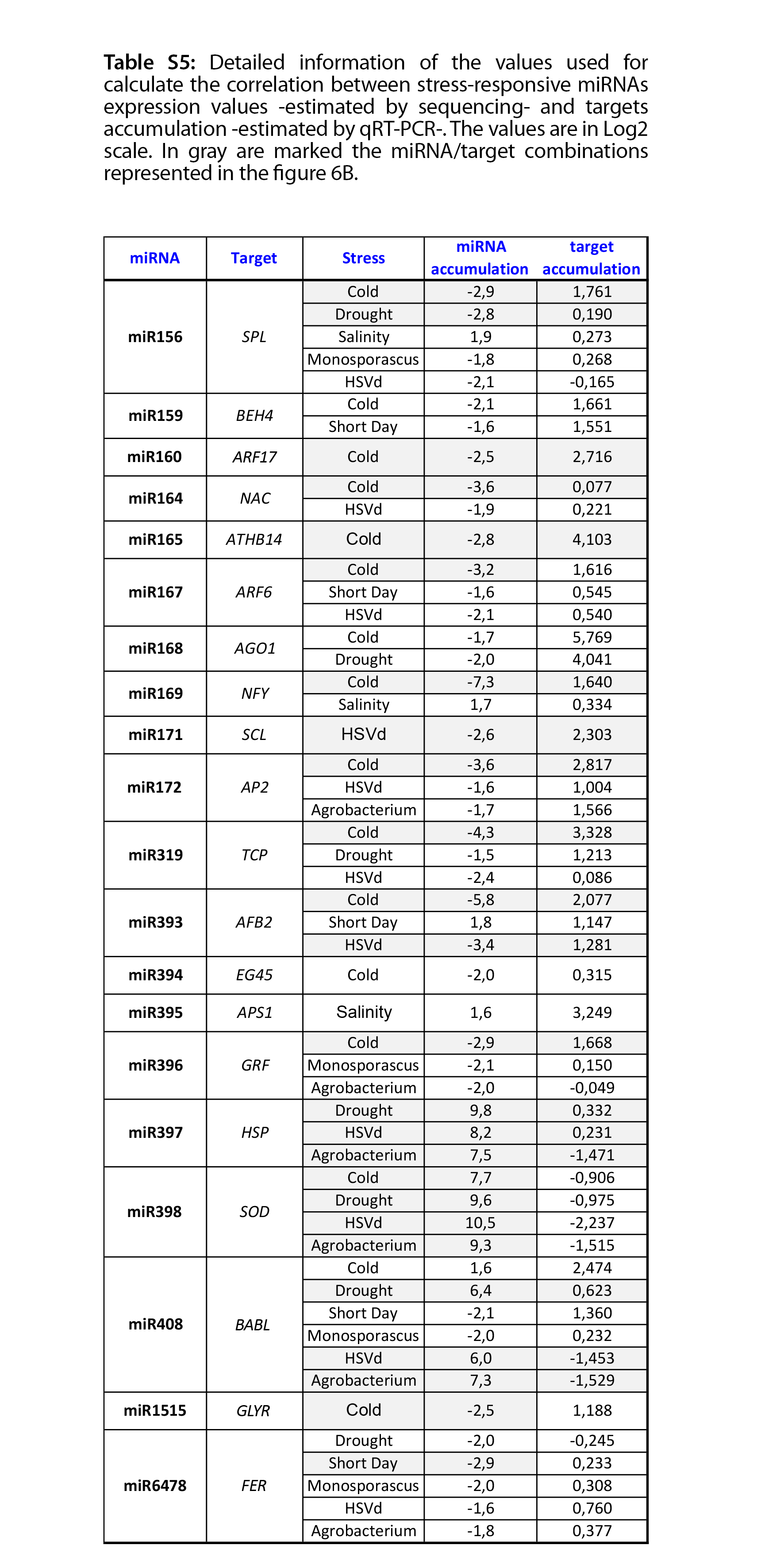

Supplement: Supplementary file 10 — Table S4. Description and detailed information of predicted targets for stress-responsive miRNAs identified en melon plants. The GO terms were estimated in base to information for homologous transcripts in arabidopsis. The putative non protein-coding TAS3 predicted as target for miR390 has not GO terms. (TIF 19740 kb) [file 12870_2019_1679_MOESM11_ESM.tif]

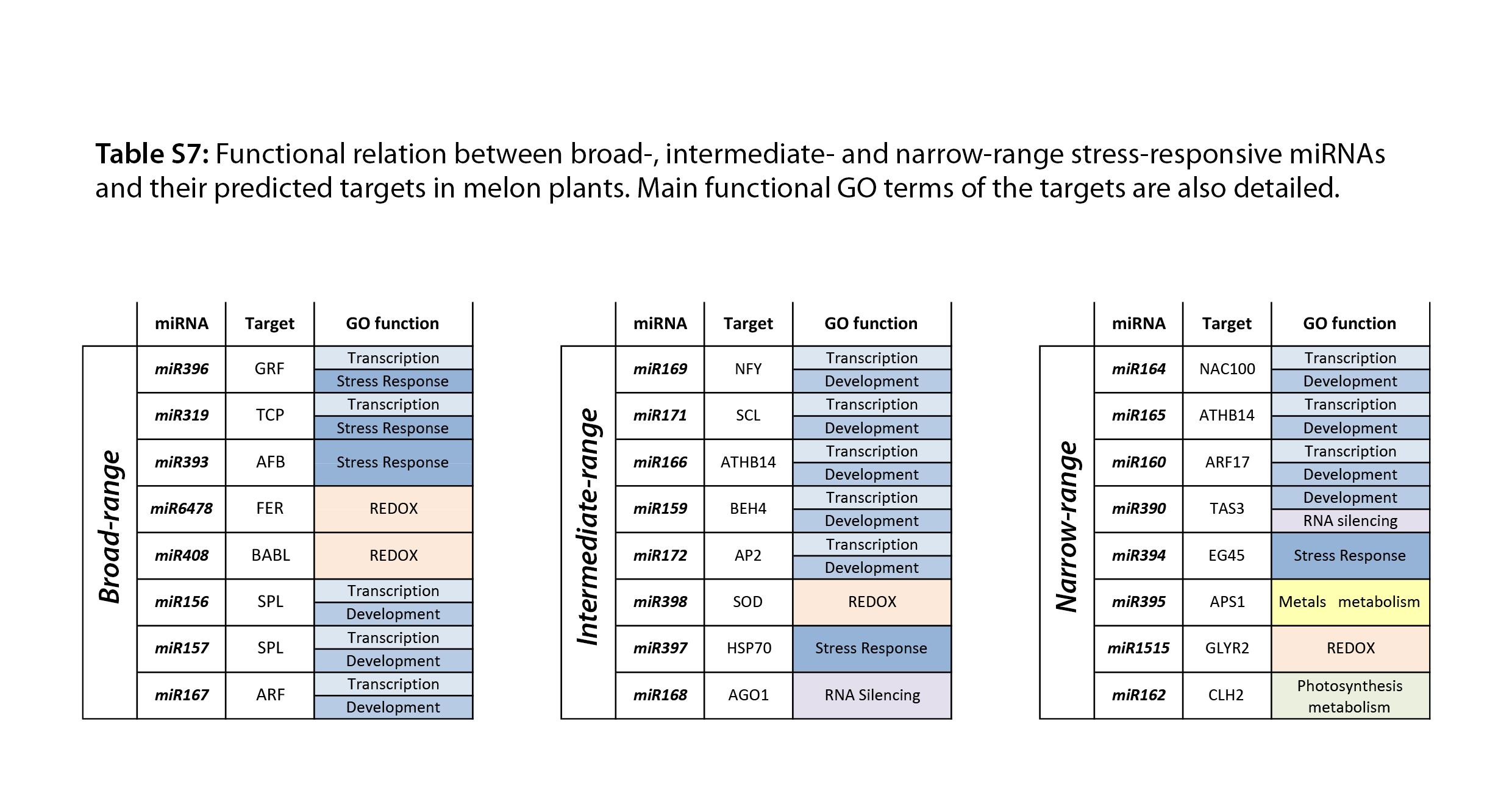

Supplement: Supplementary file 12 — Table S6. Table of presence and absence of stress-responsive miRNAs in melon plants. The values “1” and “0” represent respectively if whether or not a miRNA is responsive (with both either increased or decreased expression) to a specific stress condition. 1: stress-responsive, 0: non stress-responsive. (TIF 10502 kb) [file 12870_2019_1679_MOESM13_ESM.tif]

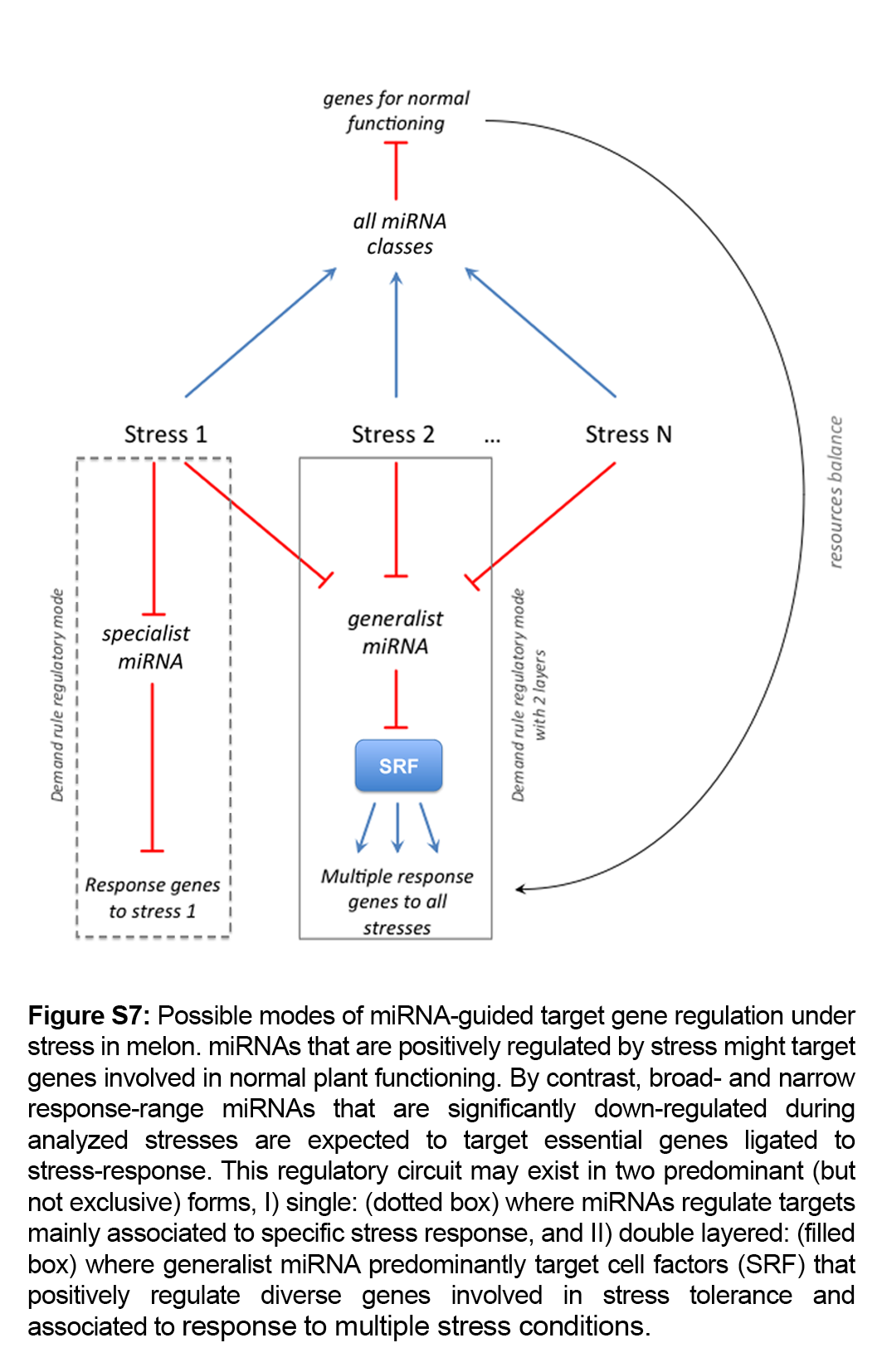

Supplement: Supplementary file 13 — Table S7. Functional relation between broad-, intermediate- and narrow-range stress-responsive miRNAs and their predicted targets in melon plants. Main functional GO terms of the targets are also detailed. (TIF 5732 kb) [file 12870_2019_1679_MOESM14_ESM.tif]

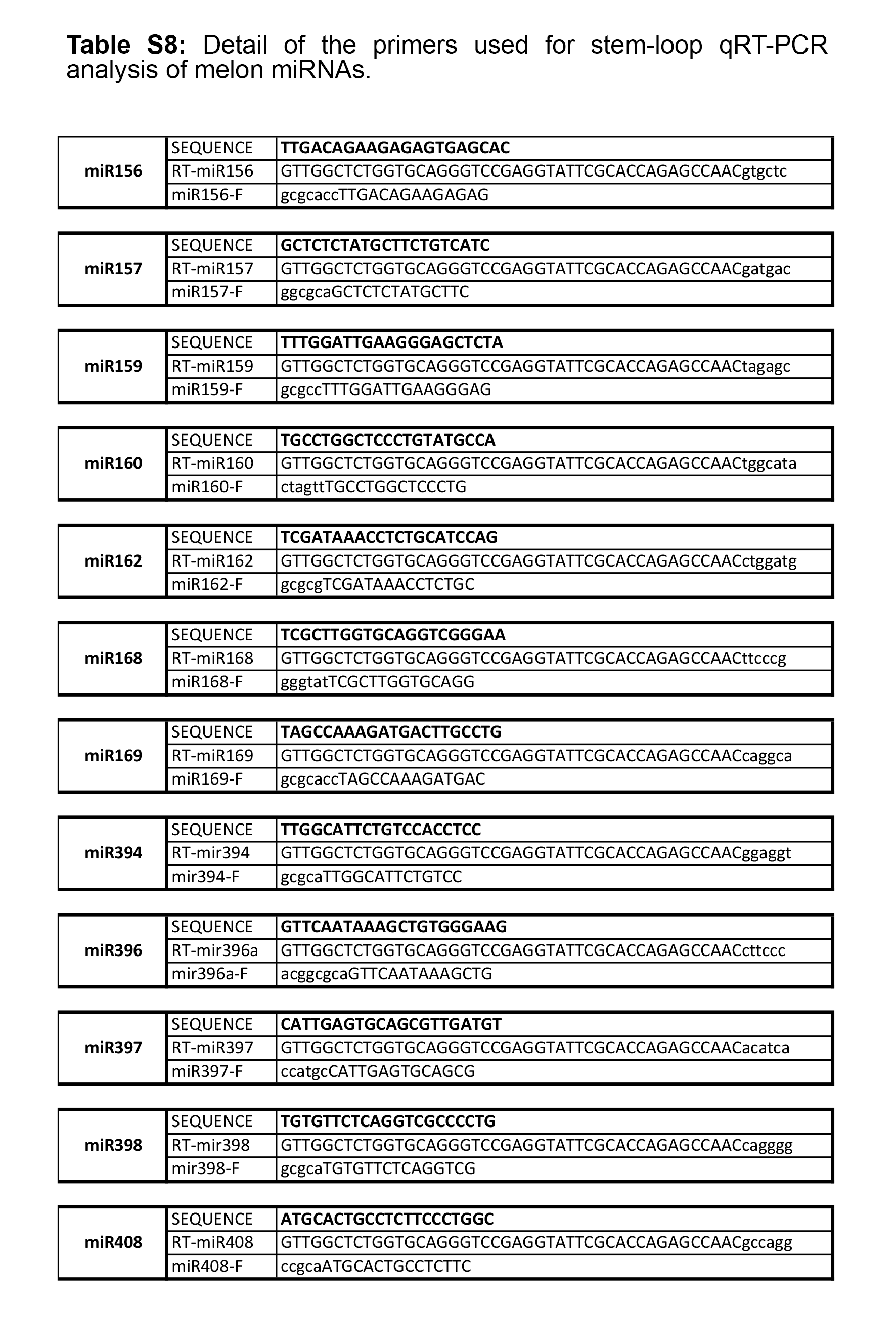

Supplement: Supplementary file 14 — Figure S7. Possible modes of miRNA-guided target gene regulation under stress in melon. miRNAs that are positively regulated by stress might target genes involved in normal plant functioning. By contrast, broad- and narrow response-range miRNAs that are significantly down-regulated during analyzed stresses are expected to target essential genes ligated to stress-response. This regulatory circuit may exist in two predominant (but not exclusive) forms, I) single: (dotted box) where miRNAs regulate targets mainly associated to specific stress response, and II) double layered: (filled box) where generalist miRNA predominantly target cell factors (SRF) that positively regulate diverse genes involved in stress tolerance and associated to response to multiple stress conditions. (TIF 15544 kb) [file 12870_2019_1679_MOESM15_ESM.tif]

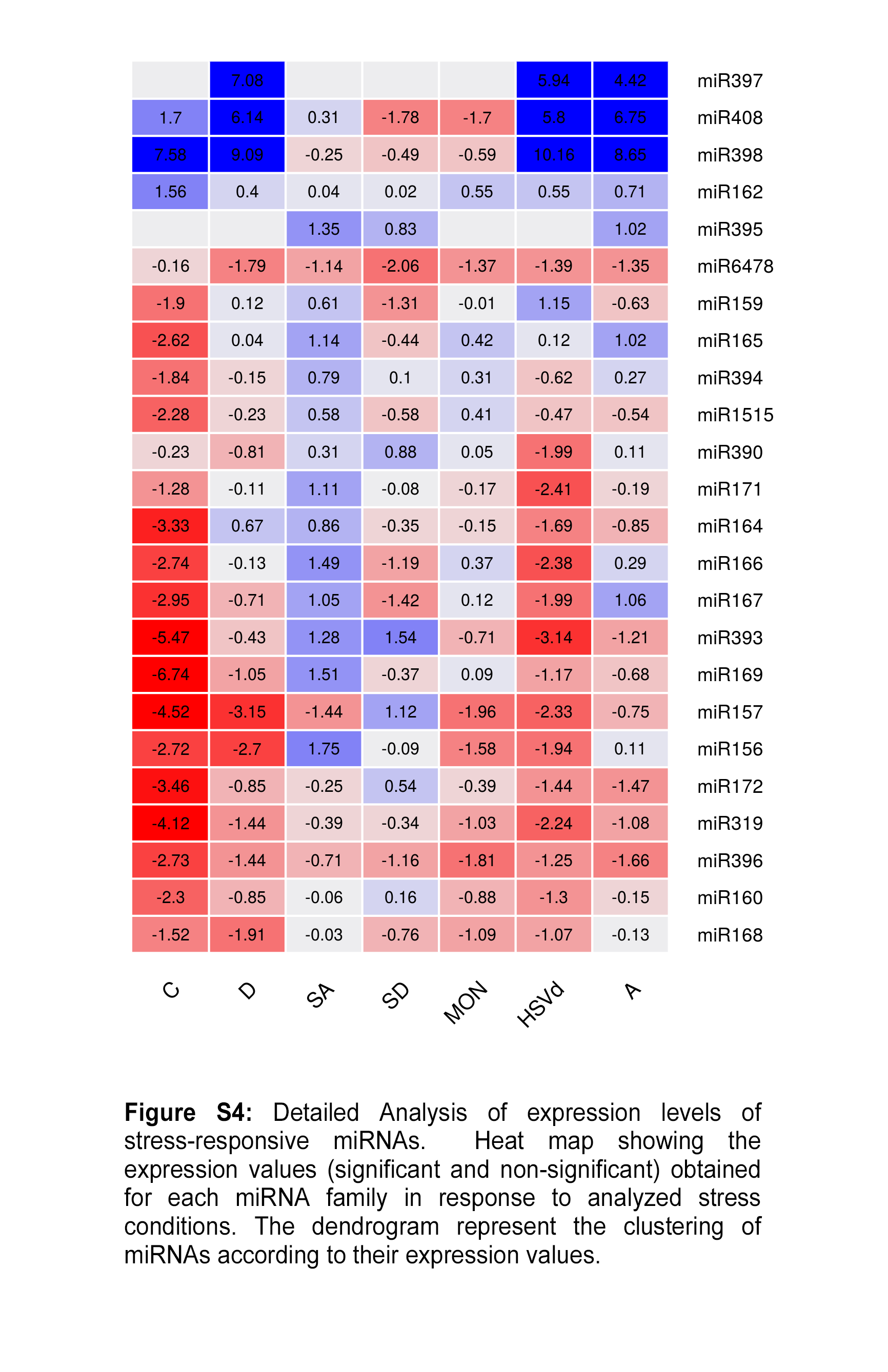

Supplement: Supplementary file 15 — Table S8. Detail of the primers used for stem-loop qRT-PCR analysis of melon miRNAs. (TIF 14782 kb) [file 12870_2019_1679_MOESM6_ESM.tif]

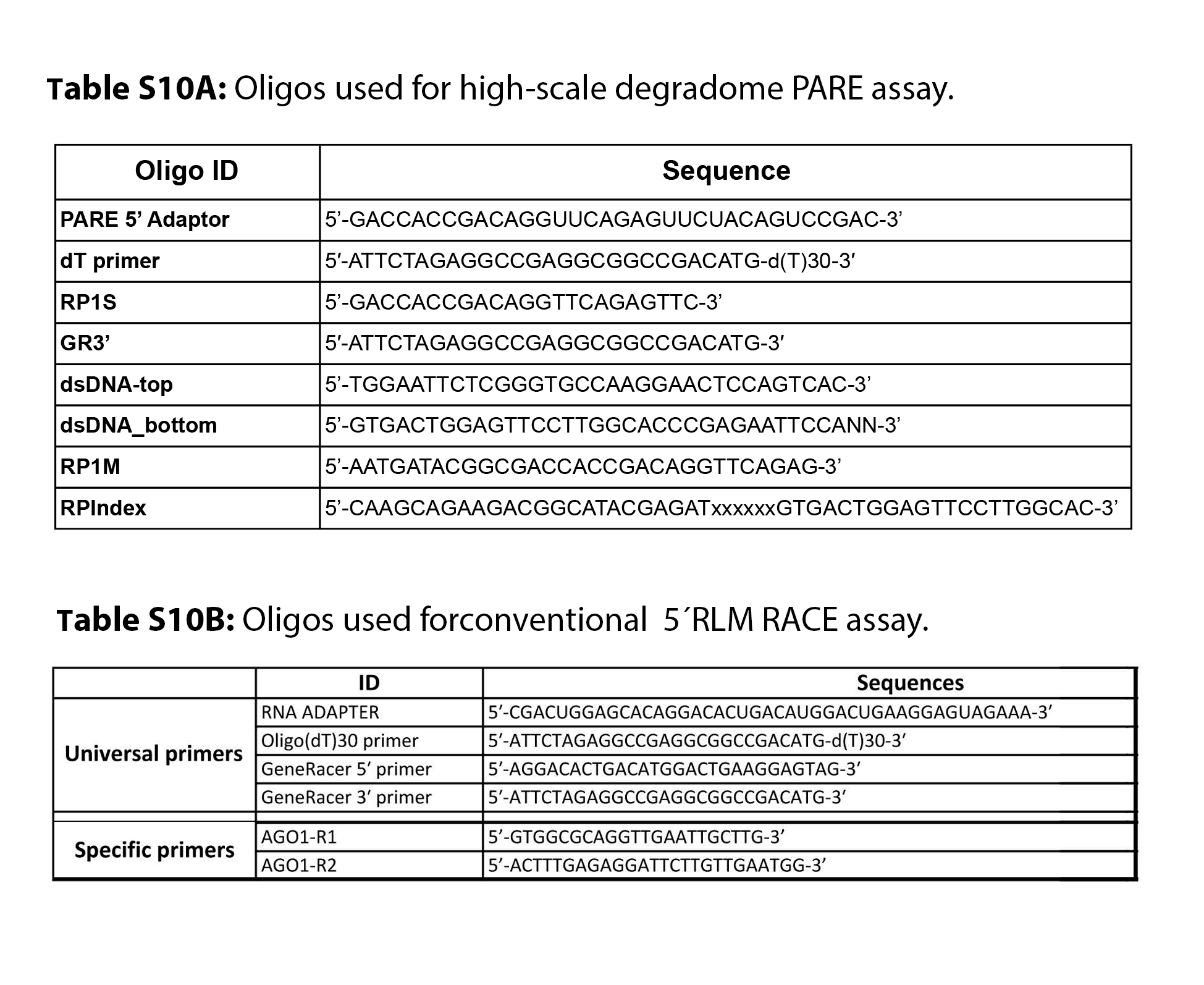

Supplement: Supplementary file 16 — Table S9. Detail of the primers used for qRT-PCR analysis of transcripts targets of stress-responsive miRNAs. (TIF 9127 kb) [file 12870_2019_1679_MOESM17_ESM.tif]

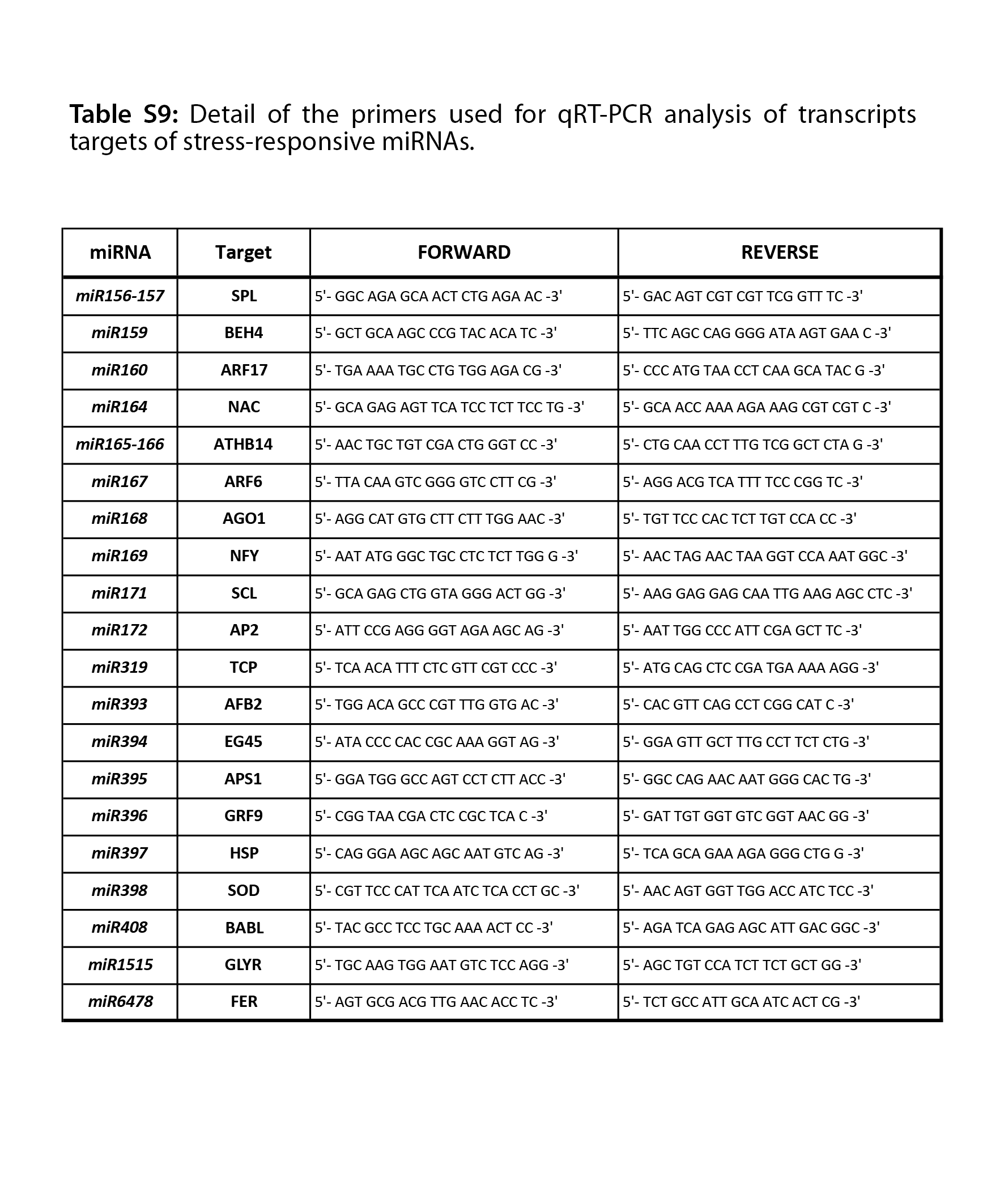

Supplement: Supplementary file 18 — Table S11. Nodes input table. Column i) indicate the name of the stress-responsive miRNA, ii) group to which they belong and iii) number of stresses in which they are present. 10: represents miRNAs responsive to 5 and 6 stress conditions, 6: represents miRNAs responsive to 3 and 4 stress conditions, and 4: represents miRNAs responsive to 1 and 2 stress conditions. (TIF 12081 kb) [file 12870_2019_1679_MOESM16_ESM.tif]
