# Supplementary material for: Inferring the regulatory network of the miRNA-mediated response to biotic and abiotic stress in melon
Source: BMC Plant Biol. 2019 Feb 18;19:78. doi: 10.1186/s12870-019-1679-0 (PMC6379984; doi:10.1186/s12870-019-1679-0)
Supplement: Supplementary file 4 — Table S2. Detail of the sRNAs sequences belonging to know miRNAs families identified as stress responsive in melón. (PDF 1429 kb) [file 12870_2019_1679_MOESM4_ESM.pdf]

Table S2: Detail of the sRNAs sequences belonging to know miRNAs families identified as stress responsive in melon.

miRNA family	sRNA	Lenght
miR156	TGACAGAAGAGAGTGAGCAC	20
	TGACAGAAGAGAGTGAGCACT	21
	TTGACAGAAGAGAGTGAGCAC	21
	TTGACAGAAGATAGAGAGCAC	21
	TTGACAGAAGATAGAGGGCAC	21
miR157	GCTCTCTATACTTCTGTCACC	21
	GCTCTCTATGCTTCTGTCATC	21
miR159	TTTGGACTGAAGGGAGCTCTA	21
	TTTGGATTGAAGGGAGCTCTC	21
	TTTGGATTGAAGGGAGCTCTG	21
	TTTGGATTGAAGGGAGCTCTT	21
miR160	TGCCTGGCTCCCTGTATGCCA	21
miR162	TCGATAAGCCTCTGCATCCAG	21
miR164	TGGAGAAGCAGGGCACGTGCT	21
miR165	TCGGACCAGGCTTCATCCCCC	21
miR166	TCGGACCAGGCTTCATTCCCC	21
	TCGGACCAGGCTTCATTCCCCC	22
	TCGGACCAGGCTTCATTCCCT	21
	TCGGACCAGGCTTCATTCCT	20
	TCGGACCAGGCTTCATTCCTC	21
	TCTCGGACCAGGCTTCATTCC	21
miR167	GGTCATGCTCTGACAGCCTCACT	23
	TGAAGCTGCCAGCATGATCT	20
	TGAAGCTGCCAGCATGATCTA	21
	TGAAGCTGCCAGCATGATCTC	21
	TGAAGCTGCCAGCATGATCTG	21
	TGAAGCTGCCAGCATGATCTT	21
	TGAAGCTGCCAGCATGATCTTA	22
miR168	CCCGCCTTGCATCAACTGAAT	21
	TCGCTTGGTGCAGGTCGGGAA	21
miR169	TAGCCAAAAATGACTTGCCTGC	22
	TAGCCAAAGATGACTTGCCTG	21
	TGAGCCAAGAATGACTTGCCGGC	23
miR171	TGATTGAGCCGCGCCAATATC	21
	TTGAGCCGCGTCAATATCTCT	21
	TTGAGCCGTGCCAATATCACG	21
miR172	AGAATCTTGATGATGCTGCAT	21
miR319	CTTGGACTGAAGGGAGCTCCC	21
	TTGGACTGAAGGGAGCTCCC	20
	TTGGACTGAAGGGAGCTCCCA	21
	TTGGACTGAAGGGAGCTCCCT	21
	TTGGACTGAAGGGAGCTCCT	20
miR390	AAGCTCAGGAGGGATAGCGCC	21
miR393	ATCATGCTATCTCTTTGGATT	21
	TCCAAAGGGATCGCATTGATC	21
miR394	TTGGCATTCTGTCCACCTCC	20
miR395	CTGAAGTGTTTGGGGGAACTC	21
miR396	GCTCAAGAAAGCTGTGGGAAA	21
	GTTCAATAAAGCTGTGGGAAA	21
	GTTCAATAAAGCTGTGGGAAG	21
	TTCAATAAAGCTGTGGGAAG	20
	TTCCACAGCTTTCTTGAACT	20
	TTCCACAGCTTTCTTGAACTA	21
	TTCCACAGCTTTCTTGAACTG	21
	TTCCACAGCTTTCTTGAACTT	21
miR397	ATTGAGTGCAGCGTTGATGT	20
miR398	CGTGTTCTCAGGTCGCCCCTG	21
	TGTGTTCCCAGGTCGCCCCTG	21
	TGTGTTCTCAGGTCACCCCT	20
	TGTGTTCTCAGGTCGCCCCCG	21
	TGTGTTCTCAGGTCGCCCCTG	21
miR408	ATGCACTGCCTCTTCCCTGGC	21
	TGCACTGCCTCTTCCCTGGC	20
miR1515	TCATTTTTGCGTGCAATGATCC	22
miR6478	CCGACCTTAGCTCAGTTGGTG	21
