# Supplementary material for: Inferring the regulatory network of the miRNA-mediated response to biotic and abiotic stress in melon
Source: BMC Plant Biol. 2019 Feb 18;19:78. doi: 10.1186/s12870-019-1679-0 (PMC6379984; doi:10.1186/s12870-019-1679-0)
Supplement: Supplementary file 11 — Table S5. Detailed information of the values used for calculate the correlation between stress-responsive miRNAs expression values -estimated by sequencing- and targets accumulation -estimated by qRT-PCR-. The values are in Log2 scale. In gray are marked the miRNA/target combinations represented in the Fig. 6b. (PDF 229 kb) [file 12870_2019_1679_MOESM12_ESM.pdf]

Table S6: Table of presence and absence of stress-responsive miRNAs in melon plants. The values “1” and “0” represent respectively if whether or not a miRNA is responsive (with both either increased or decreased expression) to a specific stress condition. 1: stress-responsive, 0: non stress-responsive.

| **miRNA** | **Cold** | **Drought** | **Sal** | **Short Day** | **Mon** | **HSVd** | **Agro** |
|---|---|---|---|---|---|---|---|
| *miR157* | 1 | 1 | 1 | 1 | 1 | 1 | 0 |
| *miR6478* | 0 | 1 | 1 | 1 | 1 | 1 | 1 |
| *miR408* | 1 | 1 | 0 | 1 | 1 | 1 | 1 |
| *miR396* | 1 | 1 | 0 | 1 | 1 | 1 | 1 |
| *miR156* | 1 | 1 | 1 | 0 | 1 | 1 | 0 |
| *miR319* | 1 | 1 | 0 | 0 | 1 | 1 | 1 |
| *miR167* | 1 | 0 | 1 | 1 | 0 | 1 | 1 |
| *miR393* | 1 | 0 | 1 | 1 | 0 | 1 | 1 |
| *miR166* | 1 | 0 | 1 | 1 | 0 | 1 | 0 |
| *miR398* | 1 | 1 | 0 | 0 | 0 | 1 | 1 |
| *miR169* | 1 | 1 | 1 | 0 | 0 | 1 | 0 |
| *miR168* | 1 | 1 | 0 | 0 | 1 | 1 | 0 |
| *miR171* | 1 | 0 | 1 | 0 | 0 | 1 | 0 |
| *miR159* | 1 | 0 | 0 | 1 | 0 | 1 | 0 |
| *miR172* | 1 | 0 | 0 | 0 | 0 | 1 | 1 |
| *miR397* | 0 | 1 | 0 | 0 | 0 | 1 | 1 |
| *miR390* | 0 | 0 | 0 | 0 | 0 | 1 | 0 |
| *miR395* | 0 | 0 | 1 | 0 | 0 | 0 | 0 |
| *miR1515* | 1 | 0 | 0 | 0 | 0 | 0 | 0 |
| *miR162* | 1 | 0 | 0 | 0 | 0 | 0 | 0 |
| *miR165* | 1 | 0 | 1 | 0 | 0 | 0 | 0 |
| *miR160* | 1 | 0 | 0 | 0 | 0 | 1 | 0 |
| *miR164* | 1 | 0 | 0 | 0 | 0 | 1 | 0 |
| *miR394* | 1 | 0 | 0 | 0 | 0 | 0 | 0 |
