# Supplementary material for: Inferring the regulatory network of the miRNA-mediated response to biotic and abiotic stress in melon
Source: BMC Plant Biol. 2019 Feb 18;19:78. doi: 10.1186/s12870-019-1679-0 (PMC6379984; doi:10.1186/s12870-019-1679-0)
Supplement: Supplementary file 17 — Table S10. Oligos used. (PDF 37 kb) [file 12870_2019_1679_MOESM18_ESM.pdf]

Table S11: Nodes input table. Column i) indicate the name of the stress-responsive miRNA, ii) group to which they belong and iii) number of stresses in which they are present. 10: represents miRNAs responsive to 5 and 6 stress conditions, 6: represents miRNAs responsive to 3 and 4 stress conditions, and 4: represents miRNAs responsive to 1 and 2 stress conditions.

name	group	size
miR157	Broad-range	10
miR6478	Broad-range	10
miR408	Broad-range	10
miR396	Broad-range	10
miR156	Broad-range	10
miR319	Broad-range	10
miR167	Broad-range	10
miR393	Broad-range	10
miR166	Intermediate-range	6
miR398	Intermediate-range	6
miR169	Intermediate-range	6
miR168	Intermediate-range	6
miR171	Intermediate-range	6
miR159	Intermediate-range	6
miR172	Intermediate-range	6
miR397	Intermediate-range	6
miR390	Narrow-range	4
miR395	Narrow-range	4
miR1515	Narrow-range	4
miR162	Narrow-range	4
miR165	Narrow-range	4
miR160	Narrow-range	4
miR164	Narrow-range	4
miR394	Narrow-range	4
