# Supplementary material for: Inferring the regulatory network of the miRNA-mediated response to biotic and abiotic stress in melon
Source: BMC Plant Biol. 2019 Feb 18;19:78. doi: 10.1186/s12870-019-1679-0 (PMC6379984; doi:10.1186/s12870-019-1679-0)
Supplement: Supplementary file 19 — Table S12. Edge input table. Values in source and target columns refers to position of the each stress responsive miRNAs in the nodes table. (PDF 25 kb) [file 12870_2019_1679_MOESM19_ESM.pdf]

Table S12. Edge input table. Values in source and target columns refers to position of the each stress responsive miRNAs in the nodes table.

source	target	value
0	1	5
0	2	5
0	3	5
0	4	5
0	5	4
0	6	4
0	7	4
0	8	4
0	9	3
0	10	4
0	11	4
0	12	3
0	13	3
0	14	2
0	15	2
0	16	1
0	17	1
0	18	1
0	19	1
0	20	2
0	21	2
0	22	2
0	23	1
1	2	5
1	3	5
1	4	4
1	5	4
1	6	4
1	7	4
1	8	3
1	9	3
1	10	3
1	11	3
1	12	2
1	13	2
1	14	2
1	15	3
1	16	1
1	17	1
1	18	0
1	19	0
1	20	1
1	21	1
1	22	1

1	23	0
2	3	6
2	4	4
2	5	5
2	6	4
2	7	4
2	8	3
2	9	4
2	10	3
2	11	4
2	12	2
2	13	3
2	14	3
2	15	3
2	16	1
2	17	0
2	18	1
2	19	1
2	20	1
2	21	2
2	22	2
2	23	1
3	4	4
3	5	5
3	6	4
3	7	4
3	8	3
3	9	4
3	10	3
3	11	4
3	12	2
3	13	3
3	14	3
3	15	3
3	16	1
3	17	0
3	18	1
3	19	1
3	20	1
3	21	2
3	22	2
3	23	1
4	5	4
4	6	3
4	7	3
4	8	3
4	9	3
4	10	4
4	11	4
4	12	3

4	13	2
4	14	2
4	15	2
4	16	1
4	17	1
4	18	1
4	19	1
4	20	2
4	21	2
4	22	2
4	23	1
5	6	3
5	7	3
5	8	2
5	9	4
5	10	3
5	11	4
5	12	2
5	13	2
5	14	3
5	15	3
5	16	1
5	17	0
5	18	1
5	19	1
5	20	1
5	21	2
5	22	2
5	23	1
6	7	5
6	8	4
6	9	3
6	10	3
6	11	2
6	12	3
6	13	3
6	14	3
6	15	2
6	16	1
6	17	1
6	18	1
6	19	1
6	20	2
6	21	2
6	22	2
6	23	1
7	8	4
7	9	3
7	10	3
7	11	2

7	12	3
7	13	3
7	14	3
7	15	2
7	16	1
7	17	1
7	18	1
7	19	1
7	20	2
7	21	2
7	22	2
7	23	1
8	9	2
8	10	3
8	11	2
8	12	3
8	13	3
8	14	2
8	15	1
8	16	1
8	17	1
8	18	1
8	19	1
8	20	2
8	21	2
8	22	2
8	23	1
9	10	3
9	11	3
9	12	2
9	13	2
9	14	3
9	15	3
9	16	1
9	17	0
9	18	1
9	19	1
9	20	1
9	21	2
9	22	2
9	23	1
10	11	3
10	12	3
10	13	2
10	14	2
10	15	2
10	16	1
10	17	1
10	18	1
10	19	1

10	20	2
10	21	2
10	22	2
10	23	1
11	12	2
11	13	2
11	14	2
11	15	2
11	16	1
11	17	0
11	18	1
11	19	1
11	20	1
11	21	2
11	22	2
11	23	1
12	13	2
12	14	2
12	15	1
12	16	1
12	17	1
12	18	1
12	19	1
12	20	2
12	21	2
12	22	2
12	23	1
13	14	2
13	15	1
13	16	1
13	17	0
13	18	1
13	19	1
13	20	1
13	21	2
13	22	2
13	23	1
14	15	2
14	16	1
14	17	0
14	18	1
14	19	1
14	20	1
14	21	2
14	22	2
14	23	1
15	16	1
15	17	0
15	18	0
15	19	0

15	20	0
15	21	1
15	22	1
15	23	0
16	17	0
16	18	0
16	19	0
16	20	0
16	21	1
16	22	1
16	23	0
17	18	0
17	19	0
17	20	1
17	21	0
17	22	0
17	23	0
18	19	1
18	20	1
18	21	1
18	22	1
18	23	1
19	20	1
19	21	1
19	22	1
19	23	1
20	21	1
20	22	1
20	23	1
21	22	2
21	23	1
22	23	1
